# A multi-species occupancy modeling approach to access the impacts of land use and land cover on terrestrial vertebrates in the Mumbai Metropolitan Region (MMR), Western Ghats, India

**DOI:** 10.1371/journal.pone.0240989

**Published:** 2020-10-21

**Authors:** Sameer Bajaru, Saunak Pal, Mrugank Prabhu, Pinal Patel, Rahul Khot, Deepak Apte

**Affiliations:** 1 Natural History Collection Department, Bombay Natural History Society, Mumbai, India; 2 Center for Environmental Research and Education (CERE), Mumbai, India; 3 Bombay Natural History Society, Mumbai, India; University of South Carolina, UNITED STATES

## Abstract

Urbanization is one of the main drivers in the conversion of natural habitats into different land use and land cover types (LULC) which threaten the local as well as global biodiversity. This impact is particularly alarming in tropical countries like India, where ~18% of the world's population live, and its ever-growing economy (i.e., industrial development) expanded urban areas by several folds. We undertook this study to examine the impacts of urbanization (i.e., LULC) on terrestrial vertebrates (mammals, birds, reptiles, and amphibians) in the Mumbai Metropolitan Region (MMR), Western Ghats, India. We sampled different habitats ranged from highly disturbed urban areas to less disturbed forested areas. Multiple sampling methods such as quadrat sampling, line transect, point count, and camera trapping were used to quantify the target taxa. We used multi-species occupancy modeling in the Bayesian framework to estimate detection probability and occupancy and to assess the effect of various LULC on different species. All four groups showed a significant negative impact of increasing anthropogenic habitat cover on occupancy. Out of 213 species detected in this study, 96% of mammals, 85% of birds, 93.75% of amphibians, and 69.43% of reptiles showed a negative effect of anthropogenic habitat cover. Evidence suggests that historical and recent human disturbances could have played an important role in transforming this area from semi-evergreen and moist deciduous forest to open, scrubby, dry deciduous, and fire-prone landscape. This might be the reason for the high occupancy of open and degraded forest habitat preferring species in our study area. We recommend species-rich areas in the MMR, e.g., Karnala Bird Sanctuary (KBS) and Prabalgad-Matheran-Malanggad Hill Range (PMMHR), must be conserved through habitat restoration, ecotourism, public awareness, and policymaking.

## Introduction

The rapid expansion of the world's urban population is a significant global driver of land use conversion and ecosystem modification [1, 2]. The urban populations are increasing with alarming rates, if the current trend continues, 6.3 billion or 70% of the global population would be inhabiting in cities by 2050, which is almost double the population of urban dwellers in 2010, 3.5 billion [3]. Unlike historical cities, modern cities are growing on an average twice as fast as urban populations [4, 5]. The most striking feature of this urban explosion is peri-urbanization, i.e., rural areas around cities are transformed into or surrounded by extended metropolitan regions [6, 7]. As a result, agriculture-forest landscapes are converted into or fragmented by urban-industrial landscapes. Peri-urbanization, the most prominent form of urban growth and urbanization in developing countries, especially in Asia and Africa, has created a complex mosaics of diverse land uses and land covers in all the ecosystems [8].

Urban growth in the coming decades will occur primarily in China, India, and Nigeria [9], and the world's 20 fastest-growing urban regions have been identified in Asia and Africa. Most of them are concentrated along coastlines and major river systems where species richness and endemism are high [10]. Many rural Asian countries are undergoing massive shifts in population trends along with economic growth, resulting in a growing percentage of the populations living in urban areas. With these growing population shifts, urbanization has become an inevitable part of economic development [11]. However, this economic development adversely affected ‘biodiversity hotspots', primarily concentrated in developing tropical countries undergoing rapid urbanization [12]. The human population growth rate in the hotspots is substantially higher than that of the global growth rate. This indicates that human-induced environmental changes in the hotspots are likely to rise by manifolds in the future, and it would further increase challenges in the conservation of these species-rich regions [10, 13]. For example, a study has forecasted that the Western Ghats of India and Sri Lanka, one of the densely populated biodiversity hotspots in the world, will largely be urbanized by 2030 [14].

The Western Ghats mountain ranges run parallel to the west coast of India (stretch over 1600 km) and are remarkably rich in species diversity and endemism: 7,000 species of flowering plants (38% endemic), 330 species of butterflies (11% endemic), 247 species of reptiles (59% endemic), 240 species of amphibians (88% endemic), 289 species of fishes (41% endemic), 508 species of birds (4% endemic) and 139 species of mammals (12% endemic) [12, 15–18]. Though forests of this region have been well explored in the past, recent discoveries of new taxa highlighted the paucity of our biodiversity knowledge and the region's potential biodiversity [19, 20]. Despite this, there has been substantial loss and degradation of the forests in the Western Ghats due to changes in land use patterns [21, 22], particularly as a result of urbanization, as some of the largest metropolitan cities like Mumbai are located in the Western Ghats.

Mumbai is located in the coastal plains of the Western Ghats. It has always been a center of development since European colonization due to its key geographic position and abundance of natural resources. Mumbai is the financial capital of the nation and the ninth populous city in the world. It has a current population of 26.6 million [23], which would grow to 44 million by 2052 and spread over 1050 km^2^, almost double the present area of 603 km^2^ [24]. There will be tremendous pressure on the already shrunken natural habitats, especially mangroves and remnant forest patches, that will eventually impact the local biodiversity [25]. As Mumbai is a coastal city, and loss of the biodiversity, especially the natural vegetation, may not only leave the city vulnerable to local environmental issues such as floods, pollutants run-off, and groundwater reduction but also global disasters like cyclones, tsunamis, and sea-level rise due to global climate change [26]. Therefore, it is crucial to know how changing land use patterns would influence the local biodiversity in this region to undertake conservation and management actions.

In biodiversity studies, individuals and communities of species (levels of organizations) are usually the focus of study, and hence a hierarchical modeling approach is necessary for understanding how land use changes affect how species communities are organized. Hierarchical models like multi-species occupancy models (MSOM), an extension of occupancy models [27], provide a framework to examine the effect of explanatory variables at multiple levels of the organization. In addition, MSOM explicitly accounts for detection probability which is usually less than one. Detection probability has overlooked in previous ecological studies, those more likely to be underestimated the abundance of species and eventually resulted in biased inferences [27–29]. The MSOM have been formulated recently, and they are increasingly used to extract information at the metacommunity level (e.g., multiple sites), community-level (single site) and, individual species level based on the occurrence (detection and non-detection) data of the multiple species recorded at different sites. This cohesive approach is based on i) distinguishing non-detection (false absence) from true absence through repetitive sampling and ii) using collective data of all species observed during sampling to improve species-specific estimates. The latter idea is useful for rare species in communities because they have too few observations to estimate their occupancy separately, and wildlife managers are particularly interested in their abundance estimation and response to various management practices [30]. Many studies have used multi-species occupancy models to understand the impacts of various habitat management practices on species and communities [30–33]. These studies also highlighted robustness, explicit parametric framework, high accuracy, low uncertainty in parameter estimation, and easy inclusion of multiple scales in MSOM.

In this study, we anticipated that species richness and occupancy of different vertebrate species would be underestimated when not corrected for detection probability, and precision of estimates would be largely affected by a low number of detections and community with a large number of rare or uncommon species (first prediction). We expected spatial congruence in species richness among all four taxonomic groups, and the forested area would be more species-rich than anthropogenic habitat covered areas (second prediction). Life-history traits such as body size, trophic guild, and mobility would influence the effect of land use and land cover on the occupancy of terrestrial vertebrates. We predicted that large-bodied species, less mobile species and habitat specialists, and dietary specialists, e.g., carnivores and frugivores, would have low occupancy and have a positive impact of increasing forest cover and negative impact of increasing anthropogenic habitat cover (third prediction).

## Methods

### Ethics statement

We conducted this study under the permission of the Maharashtra State Forest Department (Permit no. D-4/Land/258/2015-16 and D-4/Land/1949/2014-15). This study was in compliance with the laws, regulations, and procedures of this kind of biodiversity study in India, and it did not need the approval by an Institutional Animal Care and Use Committee or ethics committee.

### Study area

**Fig 1:**
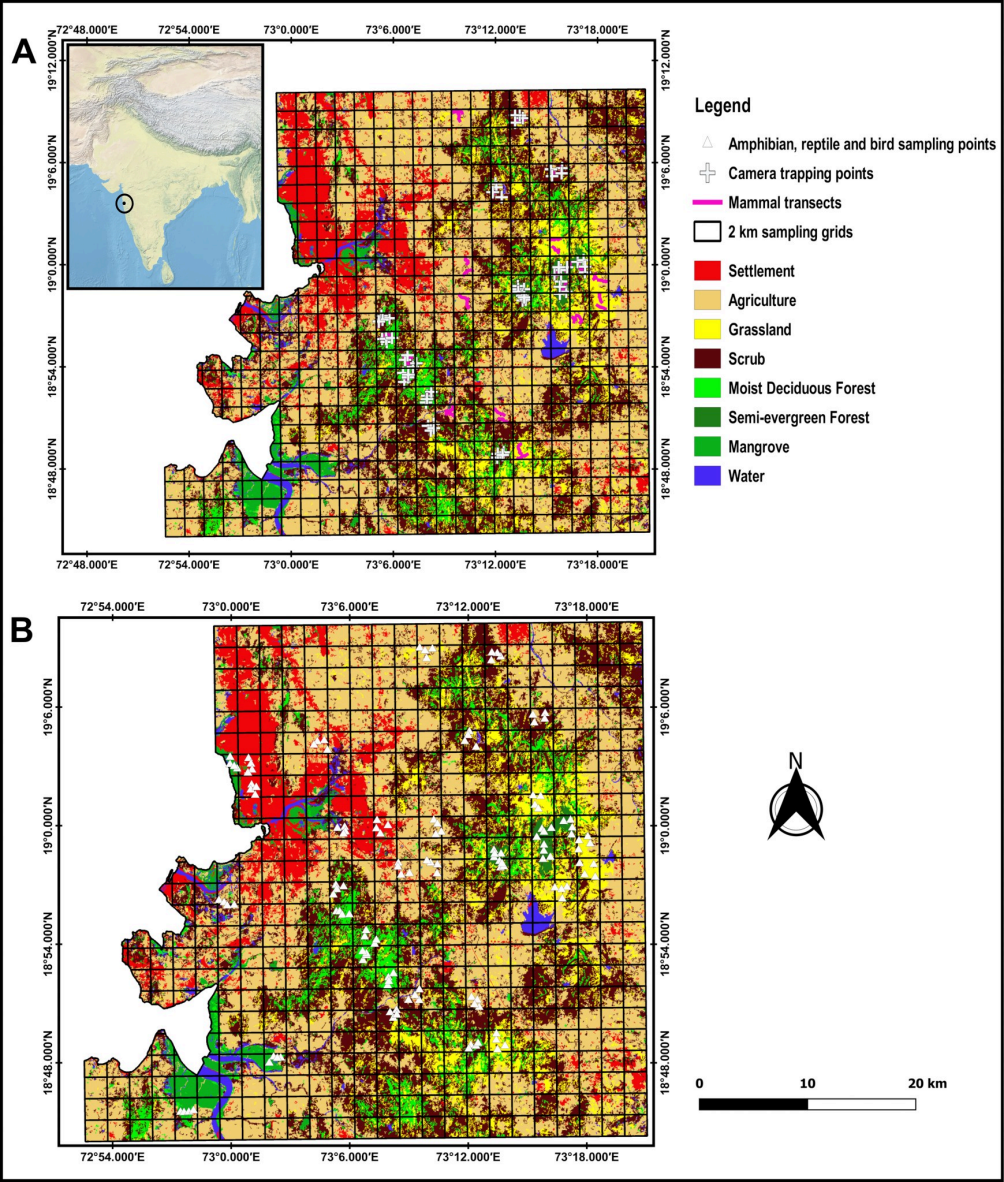
Locations of terrestrial vertebrates surveyed in the Mumbai Metropolitan Region (MMR). (A) Sampling locations of mammals, including transects and camera trapping points. The inset image shows the location of the study site in India. (B) Sampling locations of amphibians, reptiles, and birds.

This study was carried out in the eastern part of the Mumbai Metropolitan Region (MMR; 18.974845°N, 72.825741°E). The climate is tropical maritime with high humidity throughout the year and typically ranges between 44% to 76%. The average annual temperature is 27.2°C, and annual rainfall is 2167 mm [34]. Our study area (~1976 km^2^) is one of the most highly populated and developing regions in the world. It is mainly covered with agriculture (34%) followed by scrub forest (29%) and settlement (11%), so almost 2/3 of the landscape is occupied with disturbed habitats (agriculture and scrub forest; Fig 1). On the other hand, less disturbed natural habitats (primary and secondary) like moist deciduous forest (7%), semi-evergreen forest (1%), and mangrove (3%) covered only 11% of the landscape. These habitats are primarily found at different sites, e.g., mangroves at Thane, Panvel, and Karanja Creek, moist deciduous forest at Karnala Bird Sanctuary (KBS) and Manikgad, and semi-evergreen to moist deciduous forest at Prabalgad-Matheran-Malanggad Hill Range (PMMHR). The western side of the study area is coastal, flat, low elevated, and highly urbanized; in contrast, the eastern side is hilly, high elevated, and poorly urbanized. This west-east elevation gradient clearly shows a distinct pattern of distribution of land use and land cover types: low elevations (7–11m asl) are mainly covered with mangroves and mudflats; agricultural fields are located at an elevation between 16 to 48m asl; moderate elevations are occupied with scrub forests (33–200m) and moist deciduous forests and grasslands (47–365m); and high elevations (>533m) are largely covered with a semi-evergreen forest.

### Sampling design

The study area is covered with seven main habitats: semi-evergreen forest, moist deciduous forest, scrub forest, mangrove, grassland, agriculture, and settlement. The area was divided into 4-km^2^ grids for sampling mammals, birds, amphibians, and reptiles. In each habitat, we randomly selected five grids (n = 35), and in each grid, four random points situated >200m apart for sampling. These points were treated as repetitive samples for estimating detection probability of the species. We sampled these four groups, except line transect sampling for mammals, within a 100m radius of the random sampling point. All sampling was carried out between December 2015 to January 2017, with one sampling session each in winter and summer and additional session in monsoon for amphibians and reptiles. We adopted different sampling strategies for various taxa as follows.

#### Mammals

We used camera trapping and visual surveys for sampling mammals (Fig 1A). At each sampling point, an infrared camera (Cuddeback E2 Long Range IR Game/Trail Camera) was tied to the nearest tree trunk, about 40 cm above the ground using a cable lock and a metal protective case. Camera traps were placed within a 50m radius around the sampling point near animal trails, dirt roads, human paths, water bodies, fruiting trees, and other signs such as scat, pugmark, hoof mark, and feeding sign to maximize trapping success. Each grid sampled for seven consecutive nights. However, in human settlements, agriculture fields, and grasslands, we did not conduct camera trapping due to high rates of trap damage or theft.

We selected two 1-km transects in each grid for visual surveys of mammals. Forests and degraded habitats (e.g., scrub forests and grasslands), mostly the pre-existing paths were chosen, but we strictly avoided broad roads and paths frequently used by people. We walked along each transect on two consecutive days with constant speed in the morning (7.00–10.30) and the late evening to night (18.30–23.00). Mammals that were seen or heard along with indirect evidence such as scat, pellets, pugmarks, hoof marks, scratch marks, wallows, carcass, and remains of body parts were recorded.

#### Birds

We adopted a fixed-radius point count method for sampling birds [35] (Fig 1B). A 50m length was radius used to avoid the potential bias induced by varying detection probability in open habitats [36]. Point counts of 10 minutes duration were carried out on a sunny day during the first 3 hours after sunrise. All birds heard or seen within a 50m radius were recorded. All sampling was carried out by an observer familiar with birds in this region.

#### Amphibians and reptiles

Quadrats were used for sampling amphibians and reptiles (Fig 1B). Four quadrats of 25 x 25m were laid in four cardinal directions from the sampling points. The corners of each quadrat were marked with colored ribbons, and GPS locations were recorded for conducting repetitive sampling at the same place. We (two people) spent an average of 20 minutes per quadrat from late morning to afternoon (0900–1500 hrs) and from evening to night (1800–2400 hrs) searching for amphibians and reptiles. We overturned leaf litter, branches, and stones by hand, and checked rock crevices, tree holes, fissures, and buttresses for amphibians and reptiles. However, in a habitat like a mangrove, where laying cardinal quadrats was infeasible due to inaccessibility, we laid quadrats adjacent to each other (collectively 100 m length and 25 m width, comparable with an area sampled by quadrat method). Amphibians were captured by hand, while reptiles were captured using hands and snake stick. Captured individuals were released immediately after recording key morphological features used in their identification.

#### Occupancy and detection covariates

We recorded three occupancy covariates and four detection covariates that were likely to influence species occurrence in all four groups. Occupancy covariates included canopy cover, understory height, and wood stem density, whereas detection covariates included the date, time, humidity, and temperature. Understory height and wood stem density may also affect detection probability, and hence they can also be used as detection covariates. Canopy cover was measured at four cardinal directions at 100m from sampling points and 100m interval along the transects using a spherical densitometer. Wood stem density, i.e., the number of trees and shrubs (with girth at breast height more than 10cm), were recorded in two 10 x 10m plots laid 100m from the sampling point in opposite cardinal directions. Understory height (i.e., the average height of the trees and shrubs < 3m) was measured in a 1m circle around the center of amphibian and reptile sampling quadrats. Humidity and temperature were recorded using a Kestrel pocket weather meter while sampling each quadrat. In addition, we also used landscape-level covariates derived from satellite images and a digital elevation model as occupancy covariates. We classified the Landsat 8 images (LC81480472014278LGN00 and LC81480472015105LGN00) of the study area using supervised image classification for extracting the composition of various habitats around each sampling point. Similarly, ASTER GDEM (30m resolution; https://asterweb.jpl.nasa.gov/gdem.asp) was used for extracting elevation covariates around each sampling point. To avoid the complexity of the model and deal with small sample size, we pooled seven land used and land cover types into relatively homogeneous categories: semi-evergreen forest, moist deciduous forest and mangrove as a forest; scrub forest and grassland as a degraded forest; and human settlement and agriculture as an anthropogenic habitat. Finally, landscape covariates such as percentage cover of the forest, degraded forest, and human settlement, and the mean and standard deviation of elevation were extracted from the 200m buffer around sampling points.

#### Data analysis

We treated four sampling points and two transects and their temporal replicates in a grid as repetitive samples (occasions). These replicates, along with occupancy and detection covariates, were used for estimating detection and occupancy probability of the species. We pooled data across seasons (winter, summer, and monsoon) to increase the number of sampling occasions for improving occupancy estimates (i.e., accuracy and precision), as in previous studies [37–40]. We computed the correlation and variance inflation factor (VIF) for all covariates. The covariates with correlation coefficient (r) > 0.60 and VIF > 3 were discarded [41]. Finally, nine covariates (date, time, humidity, temperature, understory height, wood stem density, elevation, forest cover, and anthropogenic habitat cover) were selected for analysis. In this multi-species occupancy analysis, different variables would influence different species, and inclusion of all variables may overparameterize the models; hence, we did not include all covariates in any of the models.

MSOM were used for analyzing the data [29]. We assumed that occurrence (i.e., true presence/absence) of species *i* = 1, 2 …*N* at a site *j* = 1, 2 … *J* is a binary state denoted by *z*(*i*, *j*), where *z*(*i*, *j*) = 1 when species is present and zero otherwise. But due to imperfect detection (*p*<1), true occurrence is latent (unknown), thus it is modelled as Bernoulli distribution, i.e., *z*(*i*, *j*) ~ Bern(*Ψ*_*i*,*j*_), where *Ψ*_*i*,*j*_ is the probability of species *i* occurring at site *j*. However, what we record in field is our data *x*(*i*,*j*,*k*) for species *i* at site *j* during replicate *k* = 1,2…*K*. This observational model also follows the Bernoulli distribution *x*(*i*,*j*,*k*) ~ Bern(*p*_*i*,*j*,*k*_.*z*(*i*,*j*)), where *p*_*i*,*j*,*k*_ is detection probability of species *i* at site *j* during replicate *k*, only when *z*(*i*,*j*) = 1, if z(*i*,*j*) = 0 then *x*(*i*,*j*,*k*) = 0.

We assumed that the occurrence (*Ψ*_*i*,*j*_) and detection (*p*_*i*,*j*,*k*_) probabilities varied by species and were influenced by the habitat and survey specific factors, known as occupancy covariate and detection covariate, respectively. These effects can be incorporated into the model using the logit link function [29, 42]. Simplest model specifications are logit (*Ψ*_*i*,*j*_) = *u*_*i*_ + *α*_*j*_ (occupancy model) and logit (*θ*_*i*,*j*_) = *ʋ*_*i*_ + *β*_*j*_ (detection model), where *u*_*i*_ and *ʋ*_*i*_ are species-level effect and *α*_*j*_ and *β*_*j*_ are site-level effect.

We formulated the occupancy model as

logit (*Ψ*_*i*,*j*_) = *u*_*i*_ + *α*1_*i*_ elevation_*j*_+ *α*2_*i*_ forest cover_*j*_ + *α*3_*i*_ anthropogenic habitat cover_*j*_

Where *Ψ*_*i*,*j*_ is occurrence probability of species (*i*) at a site (*j*); *u*_*i*_ is a species-level effect; α1, α2, α3 are coefficients of occupancy covariates.

Similarly, we developed the detection probability model

logit (*θ*_*i*,*j*,*k*_) = *v*_*i*_ + *β1*_*i*_date_*j*,*k*_+ *β2*_*i*_time_*j*,*k*_ +*β3*_*i*_humidity_*j*,*k*_ + *β4*_*i*_temperature_*j*,*k*_ + *β5*_*i*_ understorey height_*j*,*k*_ + *β6*_*i*_wood stem density_*j*,*k*_

Where *θ*_*i*,*j*,*k*_ is detection probability of the species (*i*) at site (*j*) during replicate (*k*); *ʋ*_i_ is a species-level effect; and *β1* to *β6* are coefficient of detection covariates.

We adopted the Bayesian approach for estimating model parameters because hierarchical models are naturally analyzed with this approach [43]. Covariates were standardized for easy interpretation and to improve mixing and convergence of Markov Chain Monte Carlo (MCMC) chains. We followed the recommendation given particularly for prior specification in occupancy studies to avoid mistakes done in earlier studies [44, 45]. These models were run in the runjags package [46] in the program R [47]. Three parallel MCMC chains were run for 150000 iterations, and with a burn-in of the first 50000 iterations and thinning by 100, given us 3000 samples. The convergence of the MCMC chains was assessed through visual inspection of chain mixing plots and using the Gelman-Rubin statistic R-hat [48]. We calculated the Bayesian P-value as Pr (χ^2^ obs > χ^2^sim) to measure model fit—values larger than 0.95 or smaller than 0.05 indicates a lack of fit [49]. The strength of the covariate effect was determined based on the credible intervals (CI; strong effect = CI without zero, moderate effect = CI contained zero but not centered on zero, weak effect = CI centered on zero).

## Result

**Fig 2:**
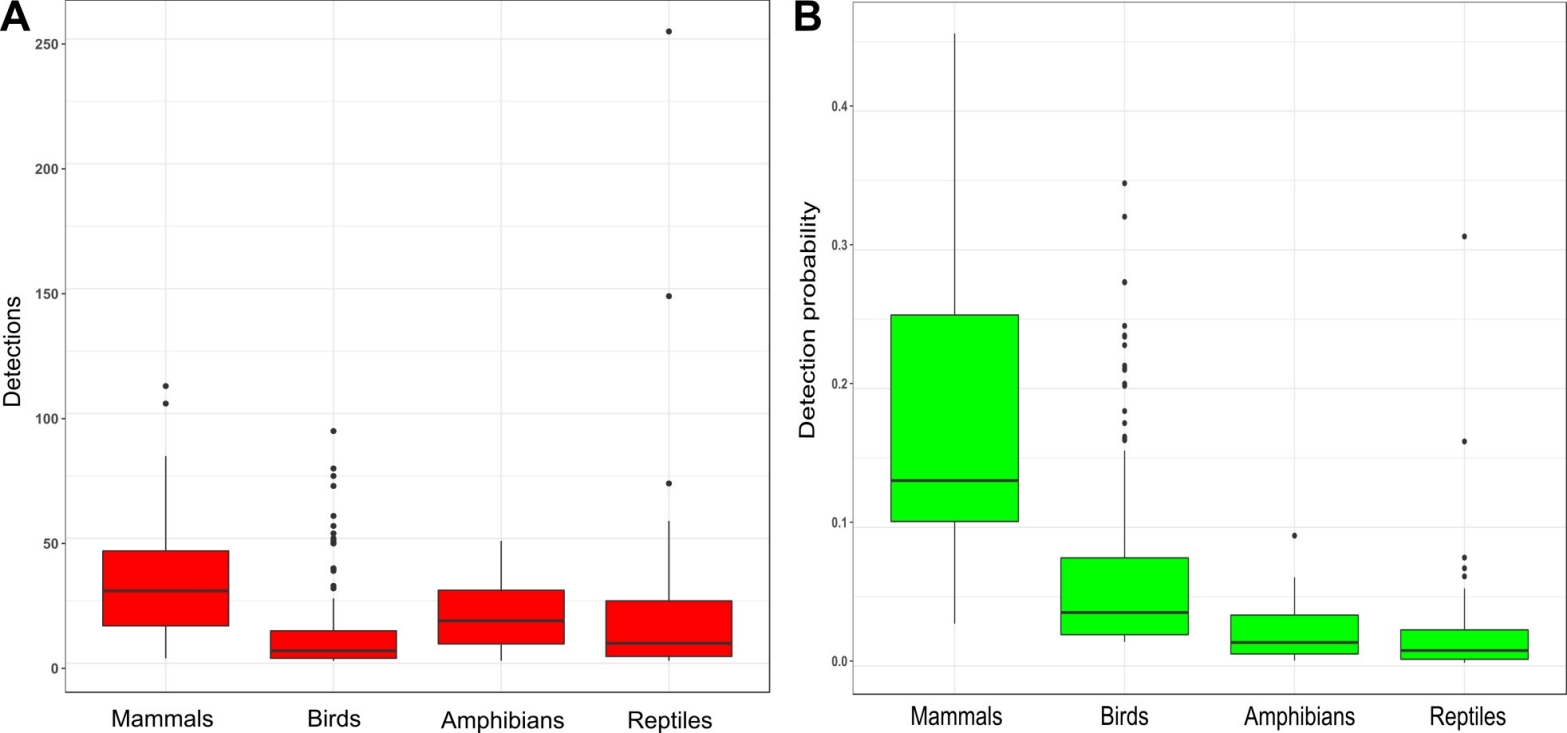
Boxplot of detections (A) and detection probability (B) of mammal, birds, amphibians, and reptiles.

In total, 213 species of terrestrial vertebrates (25 mammal species; 135 bird species; 16 amphibian species, and 36 reptile species) with 3662 detections were recorded during this study. The total number of detections varied widely among species ranged between 1 to 253 (S1 Table). In general, the number was low for most of the species—about 75% of species had less than 25 detections. Mean detection was highest for mammals and lowest for birds (Fig 2A).

### Community-level summaries and species richness

**Fig 3:**
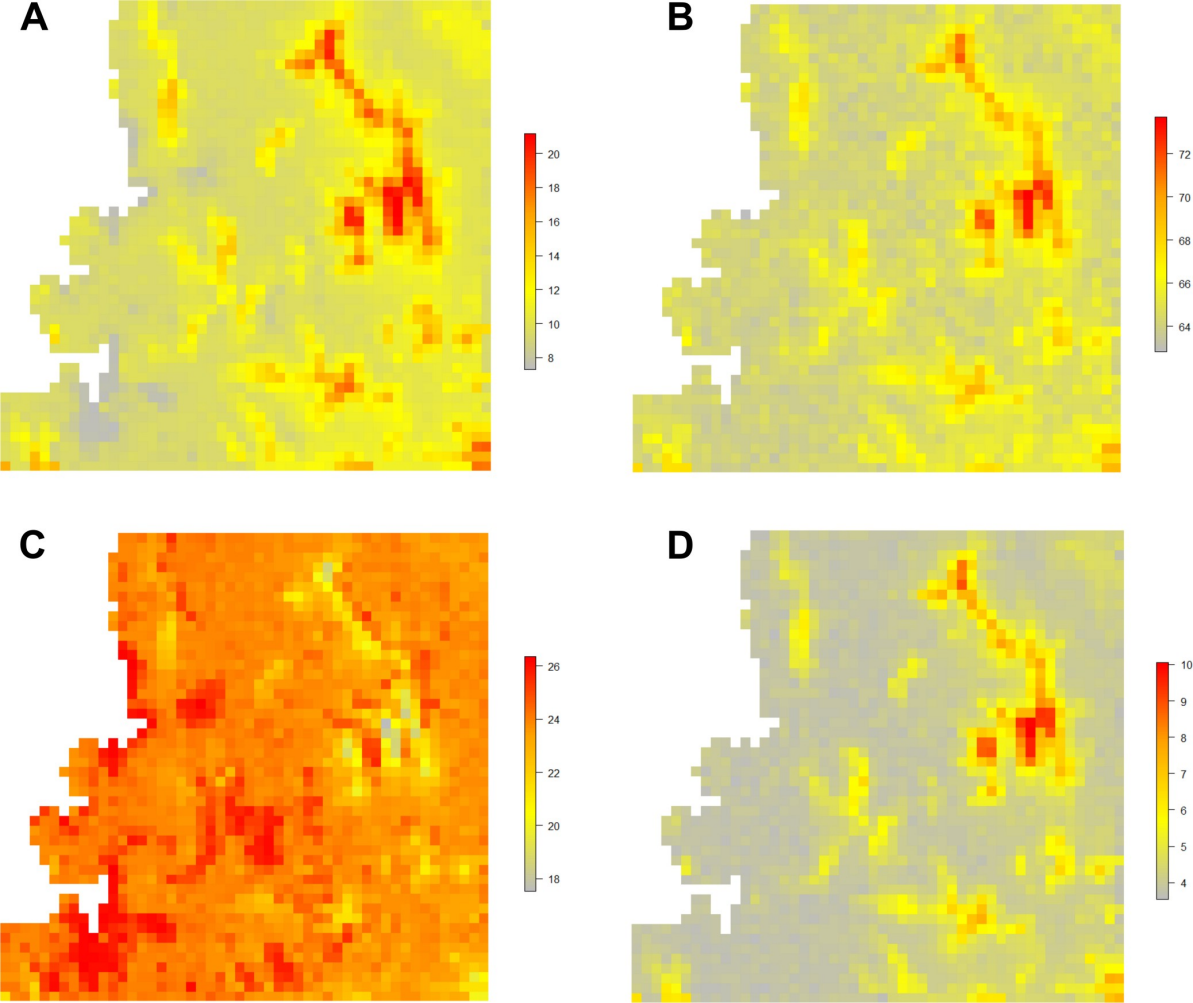
Predicted species richness of mammals (A), birds (B), reptiles (C), and amphibians (D) in MMR. Grey color indicates low species richness, and red color indicates high species richness. In mammals, birds, and amphibians, high species richness was found in the forest covered and high elevated areas; however, reptiles showed a contrasting pattern.

Occupancy of mammal and bird communities showed a negative correlation with elevation. Elevation had a significant effect (95% CI did not include 0) on birds. Amphibian and reptilian communities exhibited a moderate positive association with elevation. Overall, forest cover had a strong positive influence on mammal and amphibian species occupancy, and a weak positive effect on birds, and a moderate negative effect on reptile (Fig 3). In all four groups, species occupancy decreased significantly with increasing anthropogenic habitat cover (Table 1).

**Table 1:** Community-level summaries (mean and 95% BCI) of hyper-parameters for occupancy and detection variables for various groups.

Community-level hyper-parameter	Mammals		Birds		Amphibians		Reptiles	
	Mean	95% CI	Mean	95% CI	Mean	95% CI	Mean	95% CI
*α*1 (Elevation effect)	-0.404	-1.557, 0.665 **	-0.572	-1.081, -0.072***	0.484	-0.677, 1.713**	0.689	-0.654, 2.493**
α2 (Forest cover effect)	2.404	1.257, 3.629 ***	0.172	-0.115, 0.477*	1.311	0.353, 2.493***	-0.703	-2.289, 0.749**
α3 (Anthropogenic habitat cover effect)	-1.812	-2.866, -0.969 ***	-0.693	-1.073, -0.349***	-0.773	-1.640, -0.012***	-2.375	-4.027–1.011***
*β*1 (Date effect)	0.009	-0.097, 0.112 *	0.027	-0.060, 0.124*	-0.532	-1.364, 0.238**	-0.472	-0.582, -0.371***
*β*2 (Time effect)	-0.018	-0.094, 0.063 *	0.043	-0.022, 0.105*	0.124	-0.184, 0.402*	0.213	0.105, 0.310***
*Β*3 (Humidity effect)	-	-	-	-	0.023	-0.184, 0.241*	-	-
Β4 (Temperature effect)	-	-	-	-	-	-	-0.121	-0.237, 0.001**
*β*5 (Understorey effect)	-0.071	-0.222, 0.094 *	-0.251	-0.461, -0.054***	0.239	-0.012, 0.501**	0.421	0.235, 0.582***
*β*6 (Tree density effect)	0.080	-0.078, 0.217 *	0.038	-0.091, 0.160*	-	-	-	-

*, weak effect

**, moderate effect

***strong effect.

Overall, the influence of detection covariates on detection probability was relatively weak. However, understory had a significant effect on probability of detecting bird species (negative) and reptile species (positive). Similarly, probability of detecting reptiles decreased with the increasing date and increased with increasing time of day (Table 1).

**Fig 4:**
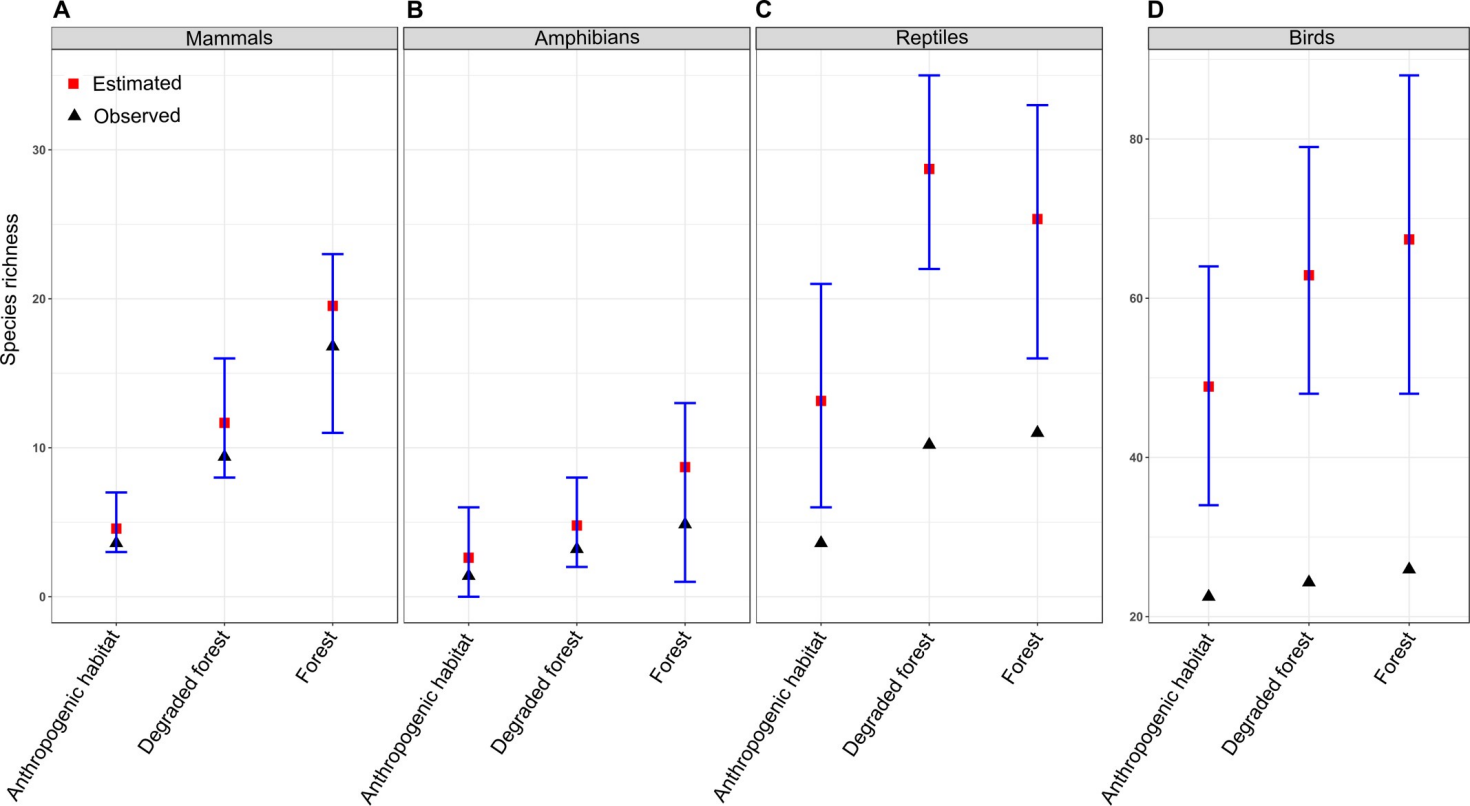
Mean estimated and observed species richness of mammals (A), amphibians (B), reptiles (C), and birds (D) in various habitats. Bars indicate 95% CI.

Observed species richness in all habitat and across all four groups was lower than that of estimated species richness, though, the underestimation was only significant for birds and reptiles (95% CI of estimated species richness did not overlap with mean observed species richness; Fig 4). Observed and estimated species richness for all groups was highest in the forest followed by degraded forest and anthropogenic habitat, except reptiles where estimated richness was highest in degraded forest. However, the estimated richness difference was only significant between forest and anthropogenic habitat for mammals.

### Species-level summaries

Occupancy of the species varied greatly across all the groups, ranged from 0.08 to 0.99. In general, detection probability was highest for mammals followed by birds, amphibians, and reptiles, though, >50% of mammals and >75% of birds, amphibians, and reptiles had detection probability <0.2 (Fig 2B). Usually, species with a very low number of detections, for example, golden jackal (*Canis aureus*), leopard (*Panthera pardus*), Indian porcupine (*Hystrix indica*), Loten's sunbird (*Cinnyris lotenius*), verditer flycatcher (*Eumyias thalassinus*), small minivet (*Pericrocotus cinnamomeus*), Bombay night frog (*Nyctibatrachus humayuni*), Battersby's caecilian (*Indotyphlus battersbyi*), Indian chameleon (*Chamaeleo zylanicus*), Indian rock python (*Python molurus*) and John's sand boa (*Eryx johnii*) had wide credible intervals for their occupancy (S1 Table).

Occupancy of the species among all the groups increased with increasing forest cover except for reptiles. However, the effect was significant among 52% of mammals (13 of 25 species) and 18.75% of amphibians (3 of 16 species), while the remaining species showed moderate to weak effect. On the other hand, 8.33% of reptiles (3 of 36 species) had a moderate negative impact of forest cover and 63.88% (23 out of 36 species) exhibited a weak negative impact. Whereas 88% of birds (118 of 135 species) depicted a moderate to weak positive effect of forest cover.

Occupancy of a large number of species across groups declined with increasing anthropogenic habitat cover. For example, 52% of mammals (13 of 25 species) showed a strong negative and 44% species (11 of 25 species) had a moderate negative impact. Similarly, 85% of bird species (115 of 135 species) showed a negative impact, of which 7% of species had a significant negative effect, and 41% of species showed a moderate negative effect. Among amphibians, except significant negative association of Bombay bush frog (*Raorchestes cf*. *bombayensis*), the remaining 15 species correlated negatively with anthropogenic habitat cover. Except, House gecko (*Hemidactylus frenatus*), 41.66% of reptiles (15 of 36 species) exhibited a moderate negative, and 27.77% (10 of 36 species) had a significant negative impact.

## Discussion

Species richness and occupancy of most of the taxa found in this study could have been underestimated if not corrected for detection probability, mainly so for communities with a large number of rare or difficult to detect species (Fig 4, S1 Table) [27–29, 38]. For example, estimated species richness was higher than observed richness for reptiles and birds (Fig 4C and 4D), probably due to rare and/or relatively hard-to-detect species in these groups [29, 50]. These findings supported our first prediction. We found that MSOM can give useful results, for instance (but not limited to), Indian giant squirrel (*Ratufa indica*) with only 13 detections at five sites had mean estimated occupancy of 0.01 (CI = 0.01–0.20). These estimates seem more credible and realistic while looking at the species habitat requirement—mature, undisturbed, and high canopy forests that are rare and restricted to higher elevations in this region.

Spatial congruence was found between species richness (for mammals, birds, and amphibians) and forest cover, i.e., high-elevation forests showed high species richness for all three groups, in accordance with the second prediction (Fig 3). On the other hand, reptile richness showed a negative correlation with elevation, and high richness was predicted in low-elevation areas with moist or deciduous forests. In general, predicted species richness was lower in areas with increasing anthropogenic habitat cover across the forest-anthropogenic habitat disturbance gradient. Other studies have reported declines in amphibian and reptile richness with increasing anthropogenic disturbances [51–53] and amphibian richness increasing with elevation [54].

Herbivorous mammals showed high occupancy, and generally increased with forest cover, as expected according to the third prediction. For instance, rhesus macaque (*Macaca mulatta*), barking deer (*Muntiacus muntjak*), wild boar (*Sus scrofa*), and Indian mouse deer (*Moschiola indica*) are largely depended on the forest for their food viz., fruits, flowers, buds, leaves, seeds and roots [55–58]. *R*. *indica* throughout its range is confined to mature forests with continuous canopy cover and high tree diversity [59]. It was further strengthened by a moderate positive correlation of this squirrel with elevation as this kind of mature forest in our study area are now confined to high-elevation areas like Matheran and Prabalgad. Similarly, the Madras treeshrew (*Anathana ellioti*) is also limited to the floor of these forests; usually, it is found away from human habitation [60]. Carnivores like Asian palm civet (*Paradoxurus hermaphroditus*), small Indian civet (*Viverricula indica*), and ruddy mongoose (*Herpestes smithii*) were also significantly associated with the forest cover. Although the former two species frequently found near the human settlements searching for rodents, fruiting trees, and dumped food; they depend on the forest cover for protection [61].

The third prediction also holds true in case of birds, for example, nectarivores, frugivores, and granivores, had low occupancy, and almost all species depicted positive association with forest cover. In general, frugivores and nectarivores have narrow food preferences (fruits and flowers), and they obtain it from undisturbed natural forests. On the other hand, omnivores and insectivores can easily find food in anthropogenically disturbed habitats due to their wide food preference [62–65]. Canopy insectivores, e.g., ashy drongo (*Dicrurus leucophaeus*), Asian paradise flycatcher (*Terpsiphone paradise*), black-naped blue monarch (*Hypothymis azurea*), brown-cheeked fulvetta (*Alcippe poioicephala*), greenish warbler (*Phylloscopus trochiloides*), heart-spotted woodpecker (*Hemicircus canente*), Malabar whistling thrush (*Myophonus horsfieldi*), orange minivet (*Pericrocotus flammeus*), puff-throated babbler (*Pellorneum ruficeps*), and white-rumped shama (*Copsychus malabaricus*) showed low occupancy and detections.

In a similar vein, omnivores like grey junglefowl (*Gallus sonneratii*), orange-headed ground thrush (*Geokichla citrine*), Indian golden oriole (*Oriolus kundoo*), and rufous treepie (*Dendrocitta vagabunda*), and frugivores and nectarivores-insectivores such as golden-fronted leafbird (*Chloropsis aurifrons*), small sunbird (*Leptocoma minima*), Nilgiri wood pigeon (*Columba elphinstonii*), and white-cheeked barbet (*Megalaima viridis*) were also had low occupancy, and moderate positive correlation with forest cover. These frugivores and nectarivores-insectivores are canopy dwelling birds. These birds mainly depend on plants belonging to families Moraceae, Lauraceae, Myristicaceae, and Loranthaceae, usually found in mature forests [65, 66]. It seems likely that the highly restricted distribution of natural and undisturbed habitats (e.g., evergreen, semi-evergreen, and riparian forests) in the study area, which these species prefer [65, 67–69], maybe the reason for their low occupancy.

Among amphibians, occupancy of *R*. cf. *bombayensis*, Ghate's bush frog (*Raorchestes ghatei*), common tree frog (*Polypedates maculatus*), and Indian dot frog (*Uperdon mormorata*) increased significantly with increasing forest cover and understory height. These species prefer semi-evergreen and moist deciduous forests, especially undergrowth below the canopy. The stream-dwelling frog *N*. *humayuni* is strictly found in high torrent streams. It had a moderate positive and negative correlation with forest and anthropogenic habitat cover, respectively. The overall response of the reptilian community was contradictory to the third prediction, mainly in the case of forest cover because most of them showed a negative association. Although, lizards such as Deccan ground gecko (*Cyrtodactylus deccanensis*) and Roux’s forest lizard (*Monilesaurus rouxii*) and snakes such as bamboo pit viper (*Trimeresurus gramineus*) and green vine snake (*Ahaetulla nasuta*) had a weak positive association with forest cover, particularly these snake species were arboreal, and ambush predators and would prefer forested habitats, especially those with substantial understory cover [70–74].

In contrast, some of the species did show a positive impact of anthropogenic habitat cover. For instance, a gecko *H*. *frenatus* showed a weak positive association with anthropogenic habitat cover, as it has in previous studies [75–77]. Occupancy of Black kite (*Milvus migrans*), blue rock pigeon (*Columba livia*), common myna (*Acridotheres tristis*), Indian pond heron (*Ardeola grayii*), red-wattled lapwing (*Vanellus indicus*), house crow (*Corvus splendens*), and house sparrow (*Passer domesticus*), increased with increasing anthropogenic habitat cover. These findings were not surprising because all these species heavily depend on food produced in human-dominated landscapes [78].

A large number of species depicted a weakly positive to moderately negative correlation with forest cover and a negative correlation with anthropogenic habitat cover. This relationship may indicate that these species prefer open or degraded habitats with relatively sparse human settlements. Hence, we anticipated moderate to high occupancy of these species as degraded forest cover (H3) is a dominant habitat in our study area. For example, Black-naped hare (*Lepus nigricollis*), one of the most abundant species (mean occupancy = 0.99, CI = 0.93–1.0) found in this study, showed a moderate negative association with anthropogenic habitat cover and weak positive correlation with forest cover. It may suggest the affinity of the species to open habitat, especially away from human settlements. Other studies also showed that Indian hares prefer areas with sparse vegetation [79, 80]. Carnivores like Indian grey mongoose (*Herpestes edwardsi*) also declined with forest cover and increased in open areas as in previous studies [81, 82]. Golden jackal, jungle cat (*Felis chaus*), and rusty-spotted cat (*Prionailurus rubiginosus*) increased slightly with forest cover and declined with increasing anthropogenic habitat cover, suggesting that these species favor disturbed or degraded forests (as in previous studies [61, 83, 84].

Omnivore birds like red-vented bulbul (*Pycnonotus cafer*), red-whiskered bulbul (*Pycnonotus jocosus*), Indian jungle crow (*Corvus culminatus*), black drongo (*Dicrurus macrocercus*), and southern coucal (*Centropus parroti*) formed one of the most frequently detected groups of birds in this study with high occupancy. This was expected according to the third prediction because these are open forest-dwelling and generalist species [62, 63, 68]. Similarly, insectivores such as plain prinia (*Prinia inornata*), ashy prinia (*Prinia socialis*), little green bee-eater (*Merops orientalis*), common iora (*Aegithina tiphia*), common tailorbird (*Orthotomus sutorius*), Indian reed-warbler (*Acrocephalus brunnescens*), and Oriental magpie robin (*Copsychus saularis*) also showed high occupancy and detection probabilities. This may be related to their preference for degraded forest habitats (e.g., scrubs and grasslands) as their occupancy decreased with increasing anthropogenic habitat cover and elevation and slightly increased with increasing forest cover. They feed on insects, a highly protein-rich food, and their small body size gives an additional advantage to survive in disturbed habitats with limited food resources.

Other studies have discussed the general pattern of the inverse relationship between body size and abundance, mass-abundance relationship [85–87]. Occupancy pattern of Asian koel (*Eudynamys scolopaceus*), brown-headed barbet (*Megalaima zeylanica*), rose-ringed parakeet (*Psittacula krameri*), and purple sunbird (*Cinnyris asiatica*) also suggest their inclination towards degraded habitats. Forest trees such as red silk-cotton (*Bombax ceiba*), flame of the forest (*Butea monosperma*), Indian fig tree (*Ficus racemosa*), Malabar plum (*Syzygium cumini*), golden shower (*Cassia fistula*), mahua (*Madhuca longifolia*), mango (*Mangifera indica*), Indian jujube (*Ziziphus mauritiana*), and Indian coral tree (*Erythrina stricta*) on which these birds feed were relatively abundant and occupied a large proportion of the study area. It may explain the prevalence of these frugivores and nectarivores in degraded forest habitats.

Occupancy of pond-dwelling frogs such as Skittering frog (*Euphlyctis cyanophlyctis*), Indian bullfrog (*Hoplobatrachus tigerinus*), and fungoid frog (*Hydrophylax bahuvistara*) was slightly increased with increasing forest cover and declined with increasing anthropogenic habitat cover. This may indicate a preference of these frogs for degraded forest cover. In such degraded habitats, ponds and puddles, usually where these frogs breed, were intentionally or accidentally created through anthropogenic disturbances, which could have facilitated their occupancy in these habitats. Murray’s house gecko *Hemidactylus* cf. *murrayi* (253 detections, mean occupancy = 0.97, CI = 0.85–0.99), the most common reptile species, showed a negative association with both forest and anthropogenic habitat cover. This species is one of the most generalistic and widely distributed among geckos and is known to occur commonly in drier and disturbed habitats such as the scrub forest, agriculture fields, and rocky outcrops [75, 76]. A similar pattern was shown by a garden lizard (*Calotes versicolor*) which is also a generalist and prefers open habitats.

In general, a high prevalence (occupancy) of species preferring degraded forest cover may indicate the degraded nature of this landscape. Our study area has a long history of disturbances. For example, two of the highly species-rich and less disturbed sites in the study area, Prabalgad and Matheran, also have gone through extensive human disturbances. Prabalgad was the main fort of Ahmednagar Kingdom in 1458 AD [88], but now it is covered with ironwood (*Memecylon umbellatum*) dominated semi-evergreen forest. Similarly, Matheran, another remnant of the semi-evergreen forest, was disturbed when it was developed as a hill station by the British in the 1850s. However, the major destruction of the landscape was carried out in the 17^th^ to the 18^th^ century, first by Portuguese and then by British colonizers for extracting timber and planting teak for the shipping industry [89]. Once covered with moist-deciduous to semi-evergreen forest, the whole area was transformed into open, dry deciduous and scrub forest. Wood extraction along with the forest fire could also have played a role in this transformation, which can be inferred from the abundance of fire-resistant trees in this area, e.g., Indian laurel (*Terminalia elliptica*), Bedda nut tree (*Terminalia bellerica*), black myrobalan (*Terminalia chebula*), flowering murdah (*Terminalia paniculata*), Kumbi (*Careya arborea*), and black catechu (*Acacia catechu*) [90].

## Conclusion and conservation recommendations

This study strengthened the importance of detection probability and multi-species occupancy modeling in monitoring species richness and occupancy, which could be otherwise underestimated. It also provided a unified framework for assessing the effect of explanatory variables on individual species as well as communities; this would facilitate the conservation biologists and wildlife managers to understand and conserve the biodiversity more quantitatively and objectively. For example, despite a low number of detections, occupancy of Indian giant squirrel was predicted precisely and accurately, and the model showed its strong association with forest cover, and it would adversely affect by anthropogenic habitat cover. It is one of the key indicator species of primary and less disturbed forest in Peninsular India; hence these quantities predictions would be crucial for management and conservation action for the long-term survival of this species.

Overall, most of the species among all four groups were negatively affected by increasing anthropogenic habitat cover. Broadly, we found three types of associations between species and habitat: a positive association with forest and negative association with anthropogenic habitat; positive association with anthropogenic habitat; and negative association with anthropogenic habitat and positive or negative association with forest cover. The last category included species preferring open habitats (excluding agriculture, an intensively modified habitat) like grasslands and scrubs; we found these were most prevalent (high occupancy) species in the study area. We anticipated as 40% of the study area is covered with degraded forest cover (scrub and grassland). Evidence suggests that human disturbances, both historic (shipping industry in the British and Portuguese colonization period) and recent (urbanization and industrialization) in MMR could have transformed semi-evergreen and moist deciduous forest dominated landscape into scrub, grasslands, and deciduous forest dominated the landscape. This could have affected the occupancy and composition of the terrestrial vertebrate in this area.

The study area is mainly covered with settlement and agriculture (51%), degraded forest (40%), and a small fraction of primary or less disturbed forest (9%). Hence, priority should be given to the conservation of remaining primary habitats and restoration of degraded habitats. Forest staff and local people should be trained to develop nurseries of native moist-deciduous or semi-evergreen trees for restoring the degraded habitats. Private landowners around species-rich areas identified in this study (e.g., KBS and PMMHR) should be encouraged to take conservation actions such as planting native trees grown in nurseries operated by forest staff and local people, and develop infrastructure for ecotourism. The Forest Department and local governing bodies like the Mumbai Metropolitan Region Development Authority (MMRDA) and the City and Industrial Development Corporation (CIDCO) may restore (and increase) the natural habitat cover in their lands or by acquiring lands. KBS and PMMHR must be protected against destructive developmental activities such as building roads, railway tracks, houses or official buildings, gardens, and playgrounds that involve the conversion of natural habitats into human-dominated habitats. These areas should be developed as wildlife tourism centers by the active participation of local stakeholders through capacity building for wildlife guides and wildlife tourism managers. We also recommend building nature interpretation centers at KBS and PMMHR for creating awareness among people about the conservation of local biodiversity of this region. In addition, state and local policymakers will be made aware of local biodiversity conservation issues by organizing meetings and workshops. MMRDA and CIDCO should provide financial support for these conservation initiatives and long-term monitoring of the impacts of urbanization on the biodiversity of MMR.

## Supporting information

S1 Table Supplemented results for multi-species occupancy of terrestrial vertebrates. Summary of the number of sites species present, naïve occupancy and number of detections; mean and 95% Bayesian credible intervals (BCI) for species-specific probabilities of occupancy and detection and effect of elevation, forest cover, and anthropogenic habitation cover in the logit scale on logit occupancy and detection. A-Mammals; B-Birds; C-Amphibians; D-Reptiles.
(DOC)

S1 File Jags code to fit the multi-species occupancy models. (TXT)

S2 File Spreadsheet of data used for the analysis of mammals. (XLSX)

S3 File Spreadsheet of data used for the analysis of birds. (XLSX)

S4 File Spreadsheet of data used for the analysis of amphibians. (XLSX)

S5 File Spreadsheet of data used for the analysis of reptiles. (XLSX)
