# Supplementary material for: A multi-species occupancy modeling approach to access the impacts of land use and land cover on terrestrial vertebrates in the Mumbai Metropolitan Region (MMR), Western Ghats, India
Source: PLoS One. 2020 Oct 21;15(10):e0240989. doi: 10.1371/journal.pone.0240989 (PMC7577471; doi:10.1371/journal.pone.0240989)
Supplement: S1 Table — Summary of the number of sites species present, naïve occupancy and number of detections; mean and 95% Bayesian credible intervals (BCI) for species-specific probabilities of occupancy and detection and effect of elevation, forest cover, and anthropogenic habitation cover in the logit scale on logit occupancy and detection. A-Mammals; B-Birds; C-Amphibians; D-Reptiles. (DOC) [file pone.0240989.s001.doc]

S1 Table. Summary of the number of sites species present, naïve occupancy and number of detections; mean and 95% Bayesian credible intervals (BCI) for species-specific probabilities of occupancy and detection and effect of elevation, forest cover and anthropogenic habitation cover in the logit scale on logit occupancy and detection.

1. **MAMMALS**

|  |  |  |  |  |  |  | **Occupancy** | | | **Detection** | | | **Slope parameter** | | |  | **Slope parameter** | | |  | **Slope parameter** | | |  |
| --- | --- | --- | --- | --- | --- | --- | --- | --- | --- | --- | --- | --- | --- | --- | --- | --- | --- | --- | --- | --- | --- | --- | --- | --- |
|  |  |  |  |  |  |  |  |  |  |  |  |  | **Elevation (α1)** | | |  | **Forest (α2)** | | |  | **Anthropogenic habitat cover (α3)** | | |  |
| **Sr. No.** | **Abbr** | **Scientific Name** | **Common Name** | **Pre** | **Naïve occupancy** | **Det** | **Mean** | **95% CI** | | **Mean** | **95% CI** | | **Mean** | **95% CI** | |  | **Mean** | **95% CI** | |  | **Mean** | **95% CI** | |  |
| 1 | B_Dr | *Muntiacus muntjak* | Barking Deer | 11 | 0.44 | 50 | 0.46 | 0.13 | 0.86 | 0.276 | 0.213 | 0.344 | -0.624 | -2.789 | 1.588 | * | 3.442 | 1.386 | 5.918 | *** | -1.816 | -3.753 | -0.233 | *** |
| 2 | B_Mq | *Macaca radiata* | Bonnet Macaque | 17 | 0.68 | 111 | 0.89 | 0.54 | 0.99 | 0.441 | 0.374 | 0.505 | -0.726 | -3.021 | 1.57 | * | 3.12 | 0.684 | 6.009 | *** | -2.233 | -4.425 | -0.94 | *** |
| 3 | BN_Hr | *Lepus nigricollis* | Black-naped Hare | 24 | 0.96 | 83 | 0.995 | 0.93 | 1 | 0.272 | 0.224 | 0.326 | -1.684 | -4.645 | 1.299 | * | 0.837 | -2.071 | 4.061 | * | -1.25 | -2.798 | 0.763 | ** |
| 4 | C_PCv | *Paradoxurus hermaphroditus* | Asian Palm Civet | 10 | 0.4 | 42 | 0.36 | 0.07 | 0.81 | 0.253 | 0.19 | 0.324 | -0.253 | -2.452 | 2.159 | * | 3.821 | 1.751 | 6.378 | *** | -1.799 | -3.966 | -0.126 | *** |
| 5 | C_PSq | *Funambulus palmarum* | Indian Palm Squirrel | 19 | 0.76 | 52 | 0.986 | 0.84 | 1 | 0.184 | 0.143 | 0.234 | -0.228 | -2.876 | 2.68 | * | 2.223 | -0.772 | 5.452 | ** | -2.131 | -4.232 | -0.712 | *** |
| 6 | F_FBt | *Rousettus leschenaulti* | Fulvous Fruit Bat | 3 | 0.12 | 8 | 0.01 | 0 | 0.18 | 0.127 | 0.053 | 0.242 | 1.16 | -0.79 | 3.684 | * | 2.021 | -0.41 | 4.887 | ** | -1.611 | -3.715 | 0.602 | ** |
| 7 | G_Jl | *Canis aureus* | Golden Jackal | 2 | 0.08 | 2 | 0.39 | 0.01 | 1 | 0.031 | 0.007 | 0.124 | -1.394 | -4.816 | 1.673 | * | 0.655 | -2.658 | 4.879 | * | -1.868 | -4.317 | -0.014 | *** |
| 8 | I_FFx | *Pteropus giganteus* | Indian Flying Fox | 16 | 0.64 | 37 | 0.95 | 0.64 | 1 | 0.14 | 0.099 | 0.187 | 0.121 | -2.461 | 3.004 | * | 2.607 | -0.123 | 5.784 | ** | -2.343 | -4.957 | -0.899 | *** |
| 9 | I_FMs | *Mus* spp. | Mice | 20 | 0.8 | 45 | 0.97 | 0.81 | 1 | 0.182 | 0.135 | 0.234 | -2.305 | -5.335 | -0.064 | * | -0.825 | -3.608 | 2.091 | * | -1.193 | -2.653 | 0.849 | ** |
| 10 | I_GMg | *Herpestes edwardsi* | Indian Grey Mongoose | 16 | 0.64 | 22 | 0.94 | 0.63 | 1 | 0.097 | 0.063 | 0.142 | -2.967 | -5.871 | -0.527 | * | -0.614 | -3.234 | 2.208 | * | -1.525 | -3.143 | 0.473 | ** |
| 11 | I_GSq | *Ratufa indica* | Indian Giant Squirrel | 5 | 0.2 | 13 | 0.01 | 0 | 0.2 | 0.151 | 0.088 | 0.238 | 2.011 | -0.018 | 4.504 | * | 2.359 | 0.035 | 5.12 | *** | -1.647 | -3.918 | 0.55 | ** |
| 12 | I_Lp | *Panthera pardus* | Indian Leopard | 4 | 0.16 | 5 | 0.03 | 0 | 0.75 | 0.06 | 0.024 | 0.132 | 1.387 | -1.429 | 4.143 | * | 2.655 | -0.136 | 6.12 | ** | -1.708 | -4.214 | 0.55 | ** |
| 13 | I_MDr | *Moschiola indica* | Indian Mouse Deer | 7 | 0.28 | 15 | 0.04 | 0 | 0.43 | 0.126 | 0.074 | 0.199 | 0.796 | -1.277 | 3.353 | * | 3.884 | 1.589 | 6.752 | *** | -1.712 | -3.825 | 0.308 | ** |
| 14 | I_Ple | *Pipistrellus* sp. | Indian Pipistrelle | 17 | 0.68 | 29 | 0.98 | 0.72 | 1 | 0.107 | 0.074 | 0.147 | 0.011 | -2.897 | 2.885 | * | 2.048 | -0.715 | 5.321 | ** | -2.416 | -4.865 | -1.004 | *** |
| 15 | I_Pn | *Hystrix indica* | Indian Porcupine | 3 | 0.12 | 3 | 0.13 | 0.01 | 0.93 | 0.042 | 0.012 | 0.117 | -1.919 | -5.16 | 1.832 | * | 2.938 | 0.296 | 6.091 | *** | -1.788 | -4.036 | 0.078 | ** |
| 16 | J_Ct | *Felis chaus* | Jungle Cat | 9 | 0.36 | 17 | 0.82 | 0.23 | 1 | 0.079 | 0.047 | 0.131 | -0.416 | -3.248 | 2.545 | * | 2.58 | -0.287 | 5.841 | ** | -2.057 | -4.336 | -0.472 | *** |
| 17 | L_BRt | *Bandicota bengalensis* | Lesser Bandicoot Rat | 17 | 0.68 | 36 | 0.98 | 0.71 | 1 | 0.134 | 0.093 | 0.187 | -1.834 | -4.725 | 0.838 | * | 0.6 | -2.07 | 4.133 | * | -1.072 | -2.659 | 1.162 | * |
| 18 | LT_TMs | *Vandeleuria oleracea* | Long-Tailed Tree Mouse | 9 | 0.36 | 16 | 0.23 | 0.02 | 0.8 | 0.107 | 0.066 | 0.161 | -0.058 | -2.563 | 2.66 | * | 4.512 | 2.07 | 7.436 | *** | -1.801 | -4.114 | 0.068 | ** |
| 19 | M_TShw | *Anathana ellioti* | Madras Treeshrew | 11 | 0.44 | 24 | 0.62 | 0.18 | 0.96 | 0.126 | 0.082 | 0.181 | -0.738 | -3.219 | 1.901 | * | 3.791 | 1.494 | 6.692 | *** | -1.725 | -3.73 | -0.065 | *** |
| 20 | R_Mg | *Herpestes smithii* | Ruddy Mongoose | 9 | 0.36 | 41 | 0.19 | 0.02 | 0.71 | 0.277 | 0.212 | 0.352 | -0.078 | -2.441 | 2.302 | * | 4.589 | 2.26 | 7.474 | *** | -1.81 | -4.136 | 0.115 | ** |
| 21 | R_Mq | *Macaca mulatta* | Rhesus Macaque | 4 | 0.16 | 31 | 0.01 | 0 | 0.12 | 0.456 | 0.334 | 0.58 | 0.156 | -1.135 | 1.578 | * | 2.286 | 0.41 | 4.617 | *** | -1.686 | -3.887 | 0.315 | ** |
| 22 | R_SCt | *Prionailurus rubiginosus* | Rusty Spotted Cat | 6 | 0.24 | 7 | 0.82 | 0.13 | 1 | 0.038 | 0.017 | 0.079 | -0.051 | -3.047 | 2.893 | * | 2.462 | -1.056 | 6.14 | * | -1.945 | -4.366 | -0.108 | *** |
| 23 | S_ICv | *Viverricula indica* | Small Indian Civet | 9 | 0.36 | 19 | 0.53 | 0.11 | 0.96 | 0.104 | 0.062 | 0.164 | -0.637 | -3.151 | 1.886 | * | 2.786 | 0.551 | 5.517 | *** | -1.834 | -4.056 | -0.147 | *** |
| 24 | NP_GLr | *Semnopithecus entellus* | Northern Plains Gray Langur | 17 | 0.68 | 104 | 0.92 | 0.57 | 0.99 | 0.425 | 0.363 | 0.489 | 0.739 | -1.64 | 3.49 | * | 2.789 | 0.356 | 5.806 | *** | -2.06 | -4.18 | -0.745 | *** |
| 25 | W_Br | *Sus scrofa* | Wild Boar | 15 | 0.6 | 55 | 0.81 | 0.4 | 0.98 | 0.231 | 0.177 | 0.291 | -0.417 | -2.64 | 1.892 | * | 2.709 | 0.495 | 5.383 | *** | -2.138 | -4.357 | -0.778 | *** |

Abbr – Abbreviation; Pre – Presence; Det – Detections; CI – Credible interval; *- Weak effect; ** - Moderate effect; *** - Strong effect

1. **BIRDS**

|  |  |  |  |  |  |  | **Occupancy** | | | **Detection** | | | **Slope parameter** | | |  | **Slope parameter** | | | | **Slope parameter** | | |  |
| --- | --- | --- | --- | --- | --- | --- | --- | --- | --- | --- | --- | --- | --- | --- | --- | --- | --- | --- | --- | --- | --- | --- | --- | --- |
|  |  |  |  |  |  |  |  |  |  |  |  |  | **Elevation (α1)** | | |  | **Forest (α2)** | | | | **Anthropogenic habitat cover (α3)** | | |  |
| **Sr. No.** | **Abbr** | **Scientific Name** | **Common Name** | **Pre** | **Naïve occupancy** | **det** | ***Mean*** | ***95% CI*** | | ***Mean*** | ***95% CI*** | | ***Mean*** | ***95% CI*** | |  | ***Mean*** | ***95% CI*** | |  | ***Mean*** | ***95% CI*** | |  |
|  |  |  |  |  |  |  |  |  |  |  |  |  |  |  |  |  |  |  |  |  |  |  |  |  |
| 1 | AS | *Tachymarptis melba* | Alpine Swift | 6 | 0.17 | 7 | 0.73 | 0.2 | 1 | 0.03 | 0 | 0.1 | 0.52 | -2.8 | 3.71 | * | -0.11 | -1.5 | 0.9 | ** | -0.41 | -2.1 | 1.22 | * |
| 2 | AD | *Dicrurus leucophaeus* | Ashy Drongo | 8 | 0.23 | 12 | 0.6 | 0.1 | 1 | 0.06 | 0 | 0.2 | 1.3 | -0.7 | 3.43 | ** | 0.26 | -0.7 | 1.3 | ** | -0.34 | -2.2 | 1.32 | * |
| 3 | AP | *Prinia socialis* | Ashy Prinia | 17 | 0.49 | 52 | 0.54 | 0.3 | 0.8 | 0.24 | 0.2 | 0.4 | -1.71 | -3.8 | 0.04 | ** | 0.13 | -0.7 | 1.1 | ** | -0.52 | -1.7 | 0.44 | ** |
| 4 | ABF | *Muscicapa dauurica* | Asian Brown Flycatcher | 1 | 0.03 | 1 | 0.12 | 0 | 0.9 | 0.03 | 0 | 0.2 | 1.21 | -1.1 | 3.69 | * | 0.27 | -0.7 | 1.5 | ** | -0.67 | -2.8 | 1.25 | * |
| 5 | AK | *Eudynamys scolopaceus* | Asian Koel | 15 | 0.43 | 22 | 0.88 | 0.5 | 1 | 0.07 | 0 | 0.1 | -1.05 | -3.9 | 2.08 | * | 0.16 | -0.9 | 1.1 | ** | -0.6 | -2.2 | 1.04 | * |
| 6 | APS | *Cypsiurus balasiensis* | Asian Palm-swift | 12 | 0.34 | 12 | 0.94 | 0.6 | 1 | 0.04 | 0 | 0.1 | -0.7 | -3.7 | 2.4 | * | 0.03 | -1.2 | 1.1 | * | -0.3 | -2 | 1.37 | * |
| 7 | AP_F | *Terpsiphone paradise* | Asian Paradise Flycatcher | 9 | 0.26 | 13 | 0.85 | 0.4 | 1 | 0.04 | 0 | 0.1 | 0.42 | -1.7 | 2.77 | * | 0.17 | -0.9 | 1.3 | ** | 0.09 | -1.8 | 1.97 | * |
| 8 | BS | *Hirundo rustica* | Barn Swallow | 4 | 0.11 | 5 | 0.4 | 0.1 | 0.9 | 0.04 | 0 | 0.1 | 0.04 | -1.8 | 2.19 | * | 0.32 | -0.6 | 1.4 | ** | -1.33 | -3.2 | 0.31 | ** |
| 9 | BD | *Dicrurus macrocercus* | Black Drongo | 23 | 0.66 | 71 | 0.74 | 0.5 | 0.9 | 0.35 | 0.3 | 0.4 | -0.67 | -1.9 | 0.37 | ** | -0.14 | -1.1 | 0.6 | ** | -0.93 | -2.1 | 0.06 | ** |
| 10 | BK | *Milvus migrans* | Black Kite | 11 | 0.31 | 16 | 0.33 | 0.1 | 0.8 | 0.1 | 0 | 0.2 | -2.52 | -5.3 | -0.2 | *** | 0.25 | -0.6 | 1.3 | ** | 0.99 | -0.4 | 2.54 | ** |
| 11 | B_HB | *Megalaima zeylanica* | Brown-headed Barbet | 11 | 0.31 | 31 | 0.25 | 0.1 | 0.6 | 0.24 | 0.1 | 0.4 | 1.27 | -0.1 | 3.21 | ** | 0.47 | -0.3 | 1.4 | ** | -1.56 | -3.4 | -0.1 | *** |
| 12 | B_HC | *Lalage melanoptera* | Black-headed Cuckooshrike | 8 | 0.23 | 13 | 0.41 | 0.1 | 0.9 | 0.09 | 0 | 0.2 | -0.76 | -3.2 | 2.19 | * | 0.13 | -0.8 | 1.1 | ** | -1.48 | -3.2 | -0.1 | *** |
| 13 | B_HI | *Threskiornis melanocephalus* | Black-headed Ibis | 2 | 0.06 | 2 | 0.29 | 0 | 0.9 | 0.02 | 0 | 0.1 | -1.65 | -4.7 | 1.36 | * | 0.25 | -0.7 | 1.3 | ** | -0.65 | -2.6 | 1.18 | * |
| 14 | B_HM | *Lonchura malacca* | Black-headed Munia | 1 | 0.03 | 1 | 0.21 | 0 | 0.9 | 0.02 | 0 | 0.1 | -1.05 | -4.4 | 2.06 | * | 0.09 | -1 | 1.1 | * | 0.07 | -1.8 | 1.99 | * |
| 15 | B_HO | *Oriolus xanthornus* | Black-hooded Oriole | 5 | 0.14 | 11 | 0.12 | 0 | 0.4 | 0.18 | 0.1 | 0.4 | -0.55 | -2.4 | 1.84 | * | 0.2 | -0.6 | 1.1 | ** | -1.62 | -3.5 | -0.2 | *** |
| 16 | B_NM | *Hypothymis azurea* | Black-naped Blue Monarch | 2 | 0.06 | 3 | 0.18 | 0 | 0.9 | 0.04 | 0 | 0.2 | 0.73 | -1.5 | 3.36 | * | 0.3 | -0.7 | 1.5 | ** | -0.99 | -3 | 0.76 | ** |
| 17 | B_WK | *Elanus caeruleus* | Black-winged Kite | 1 | 0.03 | 1 | 0.22 | 0 | 0.9 | 0.02 | 0 | 0.1 | -0.75 | -4 | 2.49 | * | 0.06 | -1.1 | 1.1 | * | -0.93 | -3 | 1.04 | * |
| 18 | B_WS | *Himantopus himantopus* | Black-winged Stilt | 1 | 0.03 | 2 | 0.09 | 0 | 0.8 | 0.05 | 0 | 0.3 | -1.27 | -4.2 | 1.4 | * | 0.03 | -1.1 | 0.9 | * | -0.73 | -2.6 | 1.1 | * |
| 19 | BRP | *Columba livia* | Blue Rock Pigeon | 12 | 0.34 | 37 | 0.23 | 0.1 | 0.6 | 0.16 | 0.1 | 0.3 | -2.54 | -5.1 | -0.3 | *** | 0.17 | -0.6 | 1 | ** | 0.73 | -0.3 | 1.82 | ** |
| 20 | B_CRT | *Monticola cinclorhyncha* | Blue-capped Rock Thrush | 4 | 0.11 | 6 | 0.35 | 0 | 0.9 | 0.04 | 0 | 0.2 | 0.73 | -1.4 | 3.3 | * | 0.18 | -0.8 | 1.2 | ** | -1.13 | -3.2 | 0.68 | ** |
| 21 | BR_W | *Acrocephalus dumetorum* | Blyth’s Reed-warbler | 2 | 0.06 | 5 | 0.05 | 0 | 0.3 | 0.16 | 0 | 0.4 | -1.56 | -4.2 | 0.61 | * | -0.05 | -1.1 | 0.8 | ** | -1.02 | -2.6 | 0.38 | ** |
| 22 | BE | *Hieraaetus pennatus* | Booted Eagle | 3 | 0.09 | 3 | 0.63 | 0.1 | 1 | 0.02 | 0 | 0.1 | -0.1 | -3.5 | 3.39 | * | 0.09 | -1 | 1.2 | * | -0.37 | -2.1 | 1.3 | * |
| 23 | B_D | *Dicrurus aeneus* | Bronze Drongo | 2 | 0.06 | 2 | 0.28 | 0 | 0.9 | 0.02 | 0 | 0.1 | -1.06 | -4.2 | 2.5 | * | 0.34 | -0.6 | 1.6 | ** | -1.15 | -3.3 | 0.79 | ** |
| 24 | B_S | *Lanius cristatus* | Brown Shrike | 2 | 0.06 | 2 | 0.34 | 0 | 1 | 0.02 | 0 | 0.1 | -1.24 | -4.2 | 2.07 | * | -0.01 | -1.3 | 1 | * | -1.01 | -3 | 1.01 | * |
| 25 | B_CF | *Alcippe poioicephala* | Brown-cheeked Fulvetta | 8 | 0.23 | 12 | 0.47 | 0.1 | 0.9 | 0.07 | 0 | 0.2 | 1.54 | -0.7 | 4.23 | ** | -0.08 | -1.3 | 0.9 | ** | -1.41 | -3.4 | 0.24 | ** |
| 26 | BHB | *Emberiza melanocephala* | Black-headed Bunting | 1 | 0.03 | 1 | 0.2 | 0 | 1 | 0.02 | 0 | 0.1 | -0.83 | -4.2 | 2.37 | * | 0.09 | -1 | 1.1 | * | 0.27 | -1.6 | 2.13 | * |
| 27 | C_W | *Motacilla citreola* | Citrine Wagtail | 1 | 0.03 | 1 | 0.22 | 0 | 1 | 0.02 | 0 | 0.1 | -1.17 | -4.4 | 1.92 | * | 0.07 | -1 | 1.1 | * | -0.22 | -2.2 | 1.82 | * |
| 28 | CH | *Upupa epops* | Common Hoopoe | 1 | 0.03 | 1 | 0.2 | 0 | 0.9 | 0.02 | 0 | 0.1 | 0.75 | -2.5 | 3.96 | * | 0.06 | -1.2 | 1.1 | * | -0.92 | -3 | 0.91 | ** |
| 29 | CI | *Aegithina tiphia* | Common Iora | 13 | 0.37 | 18 | 0.82 | 0.4 | 1 | 0.08 | 0 | 0.1 | -0.29 | -2.3 | 2.43 | * | 0.1 | -1 | 1.2 | * | -1.09 | -2.8 | 0.64 | ** |
| 30 | C_K | *Falco tinnunculus* | Common Kestrel | 1 | 0.03 | 1 | 0.24 | 0 | 0.9 | 0.02 | 0 | 0.1 | -0.67 | -3.9 | 2.68 | * | 0.07 | -1.1 | 1.1 | * | -0.95 | -3 | 0.99 | ** |
| 31 | CK | *Alcedo atthis* | Common Kingfisher | 5 | 0.14 | 5 | 0.52 | 0.1 | 1 | 0.03 | 0 | 0.1 | -0.38 | -3.1 | 2.65 | * | 0.37 | -0.5 | 1.5 | ** | -1.61 | -3.6 | 0.24 | ** |
| 32 | CM | *Acridotheres tristis* | Common Myna | 22 | 0.63 | 55 | 0.89 | 0.7 | 1 | 0.16 | 0.1 | 0.3 | -0.11 | -2.1 | 2.01 | * | 0.06 | -0.9 | 1 | * | 0.54 | -1 | 2.01 | ** |
| 33 | CR | *Fringilla coelebs* | Common Rosefinch | 4 | 0.11 | 7 | 0.28 | 0.1 | 0.9 | 0.06 | 0 | 0.2 | -1.49 | -4.3 | 1.01 | * | 0.25 | -0.7 | 1.3 | ** | -0.07 | -1.6 | 1.6 | * |
| 34 | CS | *Saxicola maurus* | Common Stonechat | 6 | 0.17 | 6 | 0.8 | 0.2 | 1 | 0.02 | 0 | 0.1 | -0.94 | -3.9 | 2.18 | * | -0.08 | -1.5 | 0.9 | * | -0.51 | -2.5 | 1.48 | * |
|  |  |  |  |  |  |  | **Occupancy** | | | **Detection** | | | **Slope parameter** | | |  | **Slope parameter** | | | | **Slope parameter** | | |  |
|  |  |  |  |  |  |  |  |  |  |  |  |  | **Elevation (α1)** | | |  | **Forest (α2)** | | | | **Anthropogenic habitat cover (α3)** | | |  |
| **Sr. No.** | **Abbr** | **Scientific Name** | **Common Name** | **Pre** | **Naïve occupancy** | **det** | ***Mean*** | ***95% CI*** | | ***Mean*** | ***95% CI*** | | ***Mean*** | ***95% CI*** | |  | ***Mean*** | ***95% CI*** | |  | ***Mean*** | ***95% CI*** | |  |
|  |  |  |  |  |  |  |  |  |  |  |  |  |  |  |  |  |  |  |  |  |  |  |  |  |
| 35 | CTB | *Orthotomus sutorius* | Common Tailorbird | 16 | 0.46 | 26 | 0.88 | 0.5 | 1 | 0.09 | 0.1 | 0.2 | -1.4 | -4.1 | 1.48 | * | 0.09 | -1 | 1.1 | * | -0.37 | -2 | 1.32 | * |
| 36 | CW | *Tephrodornis pondicerianus* | Common Woodshrike | 2 | 0.06 | 2 | 0.37 | 0 | 1 | 0.02 | 0 | 0.1 | -1.52 | -4.6 | 1.74 | * | 0.22 | -0.8 | 1.3 | ** | -0.54 | -2.5 | 1.38 | * |
| 37 | CB | *Xantholaema haemacephala* | Coppersmith Barbet | 5 | 0.14 | 5 | 0.49 | 0.1 | 1 | 0.03 | 0 | 0.1 | -1.99 | -5 | 0.84 | ** | 0.34 | -0.6 | 1.5 | ** | 0.36 | -1.3 | 2.04 | * |
| 38 | CSE | *Spilornis cheela* | Crested Serpent Eagle | 7 | 0.2 | 10 | 0.45 | 0.1 | 0.9 | 0.06 | 0 | 0.2 | 1.17 | -1.4 | 4.4 | * | 0.22 | -0.7 | 1.3 | ** | -1.46 | -3.4 | 0.19 | ** |
| 39 | DC_M | *Ptyonoprogne concolor* | Dusky Crag Martin | 2 | 0.06 | 2 | 0.36 | 0 | 0.9 | 0.02 | 0 | 0.1 | 0.64 | -2.6 | 3.97 | * | 0.03 | -1.2 | 1.1 | * | -1.22 | -3.3 | 0.6 | ** |
| 40 | ECE | *Bubulcus coromandus* | Eastern Cattel Egret | 22 | 0.63 | 48 | 0.86 | 0.6 | 1 | 0.16 | 0.1 | 0.2 | -1.56 | -4.3 | 1.06 | * | -0.26 | -1.6 | 0.7 | ** | 0.09 | -1.3 | 1.62 | * |
| 41 | ES_BD | *Anas poecilorhyncha* | Eastern Spot-billed Duck | 1 | 0.03 | 2 | 0.08 | 0 | 0.8 | 0.05 | 0 | 0.3 | -1.16 | -3.9 | 1.61 | * | 0.05 | -1.1 | 1 | * | -0.79 | -2.7 | 0.93 | ** |
| 42 | ECD | *Streptopelia decaocto* | Eurasian Collared Dove | 1 | 0.03 | 1 | 0.24 | 0 | 1 | 0.02 | 0 | 0.1 | -1.05 | -4.3 | 2.32 | * | 0.08 | -1.1 | 1.1 | * | 0.03 | -1.9 | 2.03 | * |
| 43 | ER | *Coracias garrulous* | European Roller | 1 | 0.03 | 1 | 0.23 | 0 | 0.9 | 0.02 | 0 | 0.1 | 0.52 | -2.7 | 3.74 | * | 0.08 | -1 | 1.2 | * | -0.92 | -3 | 1.02 | * |
| 44 | G_FL | *Chloropsis aurifrons* | Golden-fronted Leafbird | 10 | 0.29 | 18 | 0.35 | 0.1 | 0.8 | 0.14 | 0.1 | 0.3 | -0.55 | -2.9 | 3.35 | * | 0.1 | -0.8 | 1 | * | -2.13 | -4 | -0.7 | *** |
| 45 | GF | *Chrysocolaptes guttacristatus* | Greater Flameback | 1 | 0.03 | 1 | 0.17 | 0 | 0.9 | 0.02 | 0 | 0.1 | -0.86 | -3.9 | 2.35 | * | 0.35 | -0.6 | 1.6 | ** | -0.95 | -2.9 | 0.98 | ** |
| 46 | GRTD | *Dicrurus paradiseus* | Greater Racket-tailed Drongo | 7 | 0.2 | 9 | 0.62 | 0.1 | 1 | 0.03 | 0 | 0.1 | 0.03 | -2.1 | 3.25 | * | 0.46 | -0.5 | 1.8 | ** | -1.48 | -3.6 | 0.38 | ** |
| 47 | GW | *Phylloscopus trochiloides* | Greenish Warbler | 1 | 0.03 | 1 | 0.12 | 0 | 0.9 | 0.03 | 0 | 0.2 | 1.17 | -1.2 | 3.75 | * | 0.26 | -0.7 | 1.4 | ** | -0.68 | -2.6 | 1.18 | * |
| 48 | GJF | *Gallus sonneratii* | Grey Junglefowl | 2 | 0.06 | 2 | 0.25 | 0 | 0.9 | 0.03 | 0 | 0.2 | 0.63 | -1.6 | 3.08 | * | 0.38 | -0.6 | 1.7 | ** | -1.05 | -3.1 | 0.69 | ** |
| 49 | G_BP | *Prinia hodgsonii* | Grey-breasted Prinia | 4 | 0.11 | 6 | 0.32 | 0.1 | 0.9 | 0.05 | 0 | 0.2 | 0.5 | -2.3 | 3.54 | * | -0.12 | -1.4 | 0.9 | ** | -1.56 | -3.5 | -0 | *** |
| 50 | G_HCF | *Culicicapa ceylonensis* | Grey-headed Canary Flycatcher | 3 | 0.09 | 3 | 0.21 | 0 | 0.9 | 0.04 | 0 | 0.2 | 1.4 | -0.8 | 3.74 | ** | 0.36 | -0.6 | 1.6 | ** | -1.01 | -3.1 | 0.67 | ** |
| 51 | H_SW | *Hemicircus canente* | Heart-spotted Woodpecker | 2 | 0.06 | 3 | 0.2 | 0 | 0.9 | 0.04 | 0 | 0.2 | -1.77 | -4.6 | 0.75 | ** | 0.18 | -0.8 | 1.2 | ** | -0.47 | -2.3 | 1.33 | * |
| 52 | HC | *Corvus splendens* | House Crow | 24 | 0.69 | 78 | 0.81 | 0.5 | 1 | 0.28 | 0.2 | 0.4 | -1.72 | -3.7 | 0.25 | ** | 0.15 | -0.7 | 1.1 | ** | 0.53 | -0.8 | 1.96 | ** |
| 53 | HS | *Passer domesticus* | House Sparrow | 6 | 0.17 | 23 | 0.06 | 0 | 0.3 | 0.28 | 0.1 | 0.5 | -1.31 | -3.9 | 0.87 | ** | 0 | -1.1 | 0.8 | * | 1.32 | 0.21 | 2.59 | *** |
| 54 | IBW | *Ploceus philippinus* | Indian Baya Weaver | 1 | 0.03 | 1 | 0.22 | 0 | 0.9 | 0.02 | 0 | 0.1 | -1.15 | -4.4 | 1.97 | * | 0.08 | -1.1 | 1.1 | * | 0.1 | -1.8 | 2.05 | * |
| 55 | IBR | *Copsychus fulicatus* | Indian Black Robin | 9 | 0.26 | 11 | 0.8 | 0.3 | 1 | 0.04 | 0 | 0.1 | -1.3 | -4.4 | 1.95 | * | -0.05 | -1.4 | 1 | * | -0.13 | -1.9 | 1.67 | * |
| 56 | IC | *Phalacrocorax fuscicollis* | Indian Cormorant | 5 | 0.14 | 9 | 0.15 | 0 | 0.6 | 0.08 | 0 | 0.2 | -2.51 | -5.2 | -0 | ** | 0.29 | -0.5 | 1.3 | ** | -0.61 | -1.9 | 0.67 | ** |
| 57 | IGO | *Oriolus kundoo* | Indian Golden Oriole | 9 | 0.26 | 14 | 0.29 | 0.1 | 0.8 | 0.1 | 0.1 | 0.2 | -2.59 | -5.1 | -0.2 | ** | 0.27 | -0.6 | 1.3 | ** | -1.7 | -3.3 | -0.4 | *** |
| 58 | IGH | *Ocyceros birostris* | Indian Grey Hornbill | 1 | 0.03 | 1 | 0.18 | 0 | 0.9 | 0.02 | 0 | 0.2 | -0.88 | -4 | 2.6 | * | 0.32 | -0.6 | 1.6 | ** | -0.91 | -3.1 | 0.96 | ** |
| 59 | IJC | *Corvus culminatus* | Indian Jungle Crow | 25 | 0.71 | 50 | 0.91 | 0.7 | 1 | 0.2 | 0.2 | 0.3 | -1.01 | -2.6 | 0.82 | ** | 0.22 | -0.8 | 1.2 | ** | -0.99 | -2.6 | 0.58 | ** |
| 60 | IPH | *Ardeola grayii* | Indian Pond Heron | 10 | 0.29 | 20 | 0.21 | 0.1 | 0.6 | 0.18 | 0.1 | 0.3 | -2.85 | -5.5 | -0.7 | *** | 0.3 | -0.5 | 1.3 | ** | 0.17 | -0.9 | 1.55 | ** |
| 61 | IR_W | *Acrocephalus brunnescens* | Indian Reed-warbler | 6 | 0.17 | 20 | 0.1 | 0 | 0.3 | 0.21 | 0.1 | 0.4 | -2.59 | -5.2 | -0.4 | *** | 0.36 | -0.4 | 1.4 | ** | -1.14 | -2.4 | -0 | *** |
| 62 | I_R | *Coracias benghalensis* | Indian Roller | 1 | 0.03 | 1 | 0.22 | 0 | 1 | 0.02 | 0 | 0.1 | -0.93 | -4.1 | 2.26 | * | 0.1 | -1 | 1.2 | * | 0.27 | -1.6 | 2.12 | * |
| 63 | IME | *Egretta intermedia* | Intermediate Egret | 1 | 0.03 | 2 | 0.08 | 0 | 0.7 | 0.05 | 0 | 0.3 | -1.22 | -4.1 | 1.47 | * | 0.03 | -1.1 | 0.9 | * | -0.75 | -2.6 | 0.98 | ** |
| 64 | JB | *Turdoides striatus* | Jungle Babbler | 4 | 0.11 | 5 | 0.39 | 0.1 | 0.9 | 0.04 | 0 | 0.1 | -2.13 | -5.1 | 0.39 | ** | 0.02 | -1.1 | 1 | * | -0.86 | -2.7 | 1.06 | * |
| 65 | JP | *Prinia sylvatica* | Jungle Prinia | 5 | 0.14 | 5 | 0.61 | 0.1 | 1 | 0.03 | 0 | 0.1 | -1.39 | -4.4 | 1.81 | * | 0.02 | -1.1 | 1 | * | -1.17 | -3.1 | 0.78 | ** |
| 66 | LC | *Microcarbo niger* | Little Cormorant | 8 | 0.23 | 13 | 0.5 | 0.2 | 1 | 0.08 | 0 | 0.2 | -0.63 | -2.3 | 1.2 | * | 0.43 | -0.4 | 1.6 | ** | 0.08 | -1.6 | 1.68 | * |
| 67 | LGB | *Turdoides malcolmi* | Large Grey Babbler | 7 | 0.2 | 8 | 0.45 | 0.1 | 0.9 | 0.05 | 0 | 0.1 | -1.4 | -4.4 | 2.28 | * | -0.07 | -1.5 | 1 | * | -1.85 | -3.8 | 0.07 | ** |
| 68 | LW | *Sylvia curruca* | Lesser Whitethroat | 1 | 0.03 | 1 | 0.2 | 0 | 0.9 | 0.02 | 0 | 0.1 | -1.25 | -4.4 | 1.81 | * | 0.11 | -1 | 1.2 | * | -0.91 | -3 | 1.07 | * |
| 69 | L_C | *Coracina macei* | Large Cuckooshrike | 1 | 0.03 | 1 | 0.14 | 0 | 0.9 | 0.02 | 0 | 0.2 | 1.11 | -1.3 | 3.78 | * | 0.26 | -0.7 | 1.4 | ** | -0.66 | -2.7 | 1.27 | * |
|  |  |  |  |  |  |  | **Occupancy** | | | **Detection** | | | **Slope parameter** | | |  | **Slope parameter** | | | | **Slope parameter** | | |  |
|  |  |  |  |  |  |  |  |  |  |  |  |  | **Elevation (α1)** | | |  | **Forest (α2)** | | | | **Anthropogenic habitat cover (α3)** | | |  |
| **Sr. No.** | **Abbr** | **Scientific Name** | **Common Name** | **Pre** | **Naïve occupancy** | **det** | ***Mean*** | ***95% CI*** | | ***Mean*** | ***95% CI*** | | ***Mean*** | ***95% CI*** | |  | ***Mean*** | ***95% CI*** | |  | ***Mean*** | ***95% CI*** | |  |
|  |  |  |  |  |  |  |  |  |  |  |  |  |  |  |  |  |  |  |  |  |  |  |  |  |
| 70 | LE | *Egretta garzetta* | Little Egret | 5 | 0.14 | 8 | 0.24 | 0 | 0.8 | 0.06 | 0 | 0.2 | -2.17 | -4.8 | 0.27 | ** | 0.23 | -0.6 | 1.2 | ** | -1.17 | -2.9 | 0.33 | ** |
| 71 | LGB_E | *Merops orientalis* | Little Green Bee-eater | 22 | 0.63 | 49 | 0.77 | 0.5 | 0.9 | 0.25 | 0.2 | 0.3 | -2.76 | -4.7 | -1.2 | * | -0.03 | -1.1 | 0.9 | * | -2.07 | -3.6 | -0.8 | *** |
| 72 | LS | *Apus affinis* | Little Swift | 8 | 0.23 | 11 | 0.77 | 0.3 | 1 | 0.05 | 0 | 0.1 | 0.44 | -2.1 | 3.05 | * | -0.07 | -1.3 | 0.9 | * | -0.06 | -2 | 1.59 | * |
| 73 | L_TS | *Lanius vittatus* | Bay-backed Shrike | 1 | 0.03 | 2 | 0.11 | 0 | 0.9 | 0.04 | 0 | 0.2 | -0.61 | -3.7 | 2.48 | * | 0.12 | -1 | 1.1 | ** | 0.3 | -1.4 | 2.02 | * |
| 74 | L_S | *Cinnyris lotenius* | Loten’s Sunbird | 1 | 0.03 | 1 | 0.13 | 0 | 0.9 | 0.02 | 0 | 0.2 | 1.16 | -1.1 | 3.72 | * | 0.25 | -0.7 | 1.4 | ** | -0.66 | -2.7 | 1.31 | * |
| 75 | MWT | *Myophonus horsfieldi* | Malabar Whistling Thrush | 5 | 0.14 | 7 | 0.11 | 0 | 0.8 | 0.05 | 0 | 0.2 | 2.2 | 0.18 | 4.42 | *** | 0.31 | -0.6 | 1.4 | ** | -0.64 | -2.8 | 1.18 | * |
| 76 | NWP | *Columba elphinstonii* | Nilgiri Wood Pigeon | 4 | 0.11 | 6 | 0.1 | 0 | 0.8 | 0.05 | 0 | 0.2 | 2.04 | 0.11 | 4.32 | *** | 0.29 | -0.6 | 1.4 | ** | -0.6 | -2.6 | 1.28 | * |
| 77 | O_BP | *Anthus hodgsoni* | Olive-backed Pipit | 1 | 0.03 | 1 | 0.1 | 0 | 0.8 | 0.03 | 0 | 0.2 | 1.38 | -0.9 | 4.03 | ** | 0.26 | -0.7 | 1.4 | ** | -0.69 | -2.8 | 1.25 | * |
| 78 | OM | *Pericrocotus flammeus* | Orange Minivet | 1 | 0.03 | 1 | 0.25 | 0 | 0.9 | 0.02 | 0 | 0.1 | -0.37 | -3.7 | 3.2 | * | 0.12 | -1 | 1.2 | ** | -0.99 | -3.1 | 0.9 | ** |
| 79 | O_HGT | *Geokichla citrine* | Orange-headed Ground Thrush | 6 | 0.17 | 15 | 0.12 | 0 | 0.6 | 0.1 | 0 | 0.3 | 1.97 | 0.45 | 3.84 | *** | 0.19 | -0.8 | 1.2 | ** | -0.8 | -2.8 | 0.9 | ** |
| 80 | OHB | *Pernis ptilorhynchus* | Oriental Honey Buzzard | 3 | 0.09 | 3 | 0.62 | 0.1 | 1 | 0.02 | 0 | 0.1 | -0.31 | -3.4 | 2.85 | * | -0.01 | -1.3 | 1 | * | 0.1 | -1.8 | 2.03 | * |
| 81 | OMR | *Copsychus saularis* | Oriental Magpie Robin | 16 | 0.46 | 26 | 0.84 | 0.5 | 1 | 0.1 | 0.1 | 0.2 | -1.17 | -3.8 | 1.86 | * | 0.16 | -0.8 | 1.2 | ** | -0.91 | -2.6 | 0.74 | ** |
| 82 | OT_D | *Streptopelia orientalis* | Oriental Turtle-dove | 2 | 0.06 | 2 | 0.11 | 0 | 0.9 | 0.04 | 0 | 0.2 | 1.62 | -0.5 | 3.98 | ** | 0.3 | -0.6 | 1.5 | ** | -0.65 | -2.8 | 1.34 | * |
| 83 | PP | *Anthus rufulus* | Paddyfield Pipit | 4 | 0.11 | 5 | 0.42 | 0.1 | 0.9 | 0.04 | 0 | 0.1 | 0.63 | -2.3 | 3.63 | * | 0.34 | -0.6 | 1.5 | ** | -1.37 | -3.3 | 0.27 | ** |
| 84 | P_BF | *Dicaeum erythrorhynchos* | Pale-billed Flowerpecker | 1 | 0.03 | 1 | 0.18 | 0 | 0.9 | 0.02 | 0 | 0.1 | -1.19 | -4.3 | 1.93 | * | 0.29 | -0.6 | 1.4 | ** | -0.92 | -2.9 | 1 | * |
| 85 | P_P | *Prinia inornata* | Plain Prinia | 27 | 0.77 | 75 | 0.96 | 0.8 | 1 | 0.23 | 0.2 | 0.3 | -0.97 | -3.1 | 1.59 | * | 0.07 | -1 | 1.1 | * | -0.73 | -2.3 | 0.84 | ** |
| 86 | P_HP | *Psittacula cyanocephala* | Plum-headed Parakeet | 2 | 0.06 | 5 | 0.1 | 0 | 0.8 | 0.09 | 0 | 0.4 | -1.09 | -3.6 | 0.83 | ** | 0.17 | -0.8 | 1.1 | ** | -1.09 | -3 | 0.59 | ** |
| 87 | P_TB | *Pellorneum ruficeps* | Puff-throated Babbler | 6 | 0.17 | 13 | 0.47 | 0.1 | 1 | 0.04 | 0 | 0.1 | 0.03 | -1.8 | 1.52 | * | -0.04 | -1.3 | 1 | * | -1.08 | -3.2 | 0.91 | ** |
| 88 | PH | *Ardea purpurea* | Purple Heron | 1 | 0.03 | 1 | 0.16 | 0 | 0.9 | 0.02 | 0 | 0.1 | -1.16 | -4.1 | 1.89 | * | 0.33 | -0.7 | 1.6 | ** | -0.84 | -2.9 | 1.09 | * |
| 89 | PS | *Cinnyris asiatica* | Purple Sunbird | 28 | 0.8 | 59 | 0.97 | 0.8 | 1 | 0.2 | 0.2 | 0.3 | -0.42 | -2.2 | 1.88 | * | 0.25 | -0.7 | 1.3 | ** | -0.31 | -1.8 | 1.3 | * |
| 90 | P_RSB | *Leptocoma zeylonica* | Purple-rumped Sunbird | 5 | 0.14 | 5 | 0.71 | 0.2 | 1 | 0.03 | 0 | 0.1 | -0.29 | -2.4 | 2.1 | * | 0.42 | -0.5 | 1.7 | ** | -0.02 | -1.7 | 1.63 | * |
| 91 | RA | *Amandava amandava* | Red Avadavat | 1 | 0.03 | 1 | 0.2 | 0 | 0.9 | 0.02 | 0 | 0.1 | -1.27 | -4.3 | 1.69 | * | 0.07 | -1.1 | 1 | * | -0.81 | -2.9 | 1.07 | * |
| 92 | R_BF | *Ficedula parva* | Red-breasted Flycatcher | 5 | 0.14 | 7 | 0.18 | 0 | 0.8 | 0.06 | 0 | 0.2 | 1.75 | -0.2 | 4.26 | ** | 0.36 | -0.6 | 1.6 | ** | -0.91 | -3 | 0.84 | ** |
| 93 | R_RS | *Cecropis daurica* | Red-rumped Swallow | 2 | 0.06 | 2 | 0.3 | 0 | 0.9 | 0.02 | 0 | 0.1 | -1.08 | -4.2 | 2.14 | * | 0.25 | -0.7 | 1.4 | ** | -1.21 | -3.2 | 0.76 | ** |
| 94 | R_VB | *Pycnonotus cafer* | Red-vented Bulbul | 23 | 0.66 | 48 | 0.84 | 0.6 | 1 | 0.22 | 0.2 | 0.3 | -1.25 | -2.8 | 0.13 | ** | 0.16 | -0.7 | 1.1 | ** | -1 | -2.5 | 0.44 | ** |
| 95 | R_WL | *Vanellus indicus* | Red-wattled Lapwing | 5 | 0.14 | 9 | 0.11 | 0 | 0.5 | 0.11 | 0 | 0.3 | -1.52 | -4.3 | 0.78 | ** | -0.05 | -1.2 | 0.8 | * | 1.1 | -0.3 | 2.77 | ** |
| 96 | R_WB | *Pycnonotus jocosus* | Red-whiskered Bulbul | 33 | 0.94 | 93 | 0.98 | 0.9 | 1 | 0.32 | 0.3 | 0.4 | 0.06 | -1.9 | 2.47 | * | 0.13 | -0.9 | 1.2 | ** | -0.55 | -2.2 | 1.16 | * |
| 97 | R_RP | *Psittacula krameri* | Rose-ringed Parakeet | 11 | 0.31 | 13 | 0.86 | 0.4 | 1 | 0.06 | 0 | 0.1 | -1.62 | -4.4 | 1.72 | * | 0 | -1.2 | 0.9 | * | -0.11 | -1.7 | 1.56 | * |
| 98 | R_BC | *Porzana fusca* | Ruddy-breasted Crake | 1 | 0.03 | 1 | 0.13 | 0 | 0.9 | 0.02 | 0 | 0.1 | -1.13 | -4.2 | 2 | * | 0.37 | -0.5 | 1.6 | ** | -0.98 | -3 | 0.87 | ** |
| 99 | RT | *Dendrocitta vagabunda* | RufousTreepie | 5 | 0.14 | 8 | 0.23 | 0 | 0.8 | 0.08 | 0 | 0.2 | -1.16 | -3.9 | 2.07 | * | 0.25 | -0.6 | 1.2 | ** | -1.61 | -3.4 | -0.1 | *** |
| 100 | R_BL_TS | *Lanius schach* | Rufous-backed Shrike | 5 | 0.14 | 5 | 0.72 | 0.2 | 1 | 0.02 | 0 | 0.1 | -0.6 | -3.5 | 2.3 | * | 0.23 | -0.8 | 1.3 | ** | -1.02 | -2.9 | 0.74 | ** |
| 101 | R_TL | *Ammomanes phoenicura* | Rufous-tailed Lark | 5 | 0.14 | 7 | 0.64 | 0.1 | 1 | 0.03 | 0 | 0.1 | -0.2 | -3.3 | 2.88 | * | -0.08 | -1.4 | 0.9 | * | 0 | -1.8 | 1.74 | * |
| 102 | S_BM | *Lonchura punctulata* | Scaly-breasted Munia | 6 | 0.17 | 7 | 0.63 | 0.1 | 1 | 0.03 | 0 | 0.1 | -1.73 | -4.9 | 1.55 | * | -0.1 | -1.5 | 0.9 | * | -0.32 | -2 | 1.49 | * |
| 103 | S | *Accipiter badius* | Shikra | 10 | 0.29 | 12 | 0.7 | 0.2 | 1 | 0.05 | 0 | 0.1 | -1.25 | -3.9 | 1.52 | * | 0.18 | -0.8 | 1.2 | ** | -1.34 | -2.9 | 0.37 | ** |
| 104 | SC | *Centropus parroti* | Southern Coucal | 21 | 0.6 | 38 | 0.87 | 0.6 | 1 | 0.17 | 0.1 | 0.2 | -0.93 | -2.8 | 1.76 | * | 0.17 | -0.8 | 1.3 | ** | -1.12 | -2.7 | 0.42 | ** |
| 105 | SM | *Taccocua leschenultii* | Sirkeer Malkoha | 4 | 0.11 | 5 | 0.54 | 0.1 | 1 | 0.04 | 0 | 0.2 | 0.31 | -1.9 | 2.94 | * | 0.24 | -0.7 | 1.4 | ** | -0.49 | -2.3 | 1.14 | * |
|  |  |  |  |  |  |  | **Occupancy** | | | **Detection** | | | **Slope parameter** | | |  | **Slope parameter** | | | | **Slope parameter** | | |  |
|  |  |  |  |  |  |  |  |  |  |  |  |  | **Elevation (α1)** | | |  | **Forest (α2)** | | | | **Anthropogenic habitat cover (α3)** | | |  |
| **Sr. No.** | **Abbr** | **Scientific Name** | **Common Name** | **Pre** | **Naïve occupancy** | **det** | ***Mean*** | ***95% CI*** | | ***Mean*** | ***95% CI*** | | ***Mean*** | ***95% CI*** | |  | ***Mean*** | ***95% CI*** | |  | ***Mean*** | ***95% CI*** | |  |
|  |  |  |  |  |  |  |  |  |  |  |  |  |  |  |  |  |  |  |  |  |  |  |  |  |
| 106 | S_M | *Pericrocotus cinnamomeus* | Small Minivet | 4 | 0.11 | 4 | 0.69 | 0.1 | 1 | 0.02 | 0 | 0.1 | 0.19 | -2.2 | 2.99 | * | 0.26 | -0.8 | 1.4 | ** | -0.49 | -2.4 | 1.3 | * |
| 107 | SS | *Leptocoma minima* | Small Sunbird | 3 | 0.09 | 4 | 0.39 | 0 | 1 | 0.03 | 0 | 0.1 | 0.37 | -2.2 | 3.37 | * | 0.32 | -0.6 | 1.6 | ** | -1.18 | -3.3 | 0.66 | ** |
| 108 | S_C | *Phyloscopus tristis* | Siberian Chiffchaff | 4 | 0.11 | 5 | 0.27 | 0 | 0.9 | 0.04 | 0 | 0.1 | -2.15 | -5.1 | 0.85 | ** | 0.3 | -0.6 | 1.4 | ** | -1.02 | -2.8 | 0.72 | ** |
| 109 | SGS | *Laniusmeridionalis* | Southern Grey Shrike | 3 | 0.09 | 3 | 0.5 | 0.1 | 1 | 0.02 | 0 | 0.1 | -1.54 | -4.8 | 1.61 | * | 0.01 | -1.2 | 1 | * | -0.09 | -2 | 1.85 | * |
| 110 | SD | *Streptopelia chinensis* | Spotted Dove | 17 | 0.49 | 38 | 0.51 | 0.2 | 0.8 | 0.2 | 0.1 | 0.3 | -2.97 | -5.6 | -1 | *** | 0.1 | -0.8 | 1 | ** | -1.03 | -2.2 | 0.27 | ** |
| 111 | S_TB | *Hypsipetes ganeesa* | Square-tailed Bulbul | 3 | 0.09 | 7 | 0.1 | 0 | 0.8 | 0.04 | 0 | 0.2 | 1.61 | -0.4 | 4 | ** | 0.27 | -0.7 | 1.4 | ** | -0.6 | -2.6 | 1.28 | * |
| 112 | SE | *Aquila nipalensis* | Steppe Eagle | 1 | 0.03 | 1 | 0.17 | 0 | 0.9 | 0.02 | 0 | 0.1 | -0.79 | -4.1 | 2.57 | * | 0.37 | -0.6 | 1.6 | ** | -0.93 | -3 | 0.83 | ** |
| 113 | SW | *Iduna rama* | Sykes's Warbler | 2 | 0.06 | 2 | 0.2 | 0 | 0.9 | 0.02 | 0 | 0.1 | -1.49 | -4.4 | 1.45 | * | 0.46 | -0.4 | 1.8 | ** | -0.98 | -3.1 | 0.89 | ** |
| 114 | T_BF | *Dicaeum agile* | Thick-billed Flowerpecker | 4 | 0.11 | 6 | 0.27 | 0 | 0.9 | 0.06 | 0 | 0.2 | 0.41 | -1.7 | 3.03 | * | 0.35 | -0.5 | 1.4 | ** | -1.29 | -3.2 | 0.31 | ** |
| 115 | TBF | *Cyornis tickelliae* | Tickell's Blue Flycatcher | 5 | 0.14 | 5 | 0.76 | 0.2 | 1 | 0.02 | 0 | 0.1 | -0.49 | -2.5 | 2.12 | * | 0.26 | -0.8 | 1.4 | ** | -0.61 | -2.4 | 1.23 | * |
| 116 | TP | *Anthus trivialis* | Tree Pipit | 3 | 0.09 | 4 | 0.41 | 0.1 | 1 | 0.03 | 0 | 0.1 | -1.06 | -4.2 | 1.97 | * | 0 | -1.2 | 1 | * | -0.57 | -2.3 | 1.19 | * |
| 117 | UI | *Phylloscopus* sp. | UI Warbler | 2 | 0.06 | 2 | 0.45 | 0 | 1 | 0.02 | 0 | 0.1 | -1.11 | -4.5 | 2.18 | * | 0.03 | -1.3 | 1.1 | * | -0.41 | -2.4 | 1.38 | * |
| 118 | VF | *Eumyias thalassinus* | Verditer Flycatcher | 1 | 0.03 | 2 | 0.07 | 0 | 0.8 | 0.04 | 0 | 0.3 | 0.96 | -1.1 | 3.57 | * | 0.23 | -0.8 | 1.3 | ** | -0.62 | -2.7 | 1.3 | * |
| 119 | VS | *Aethopyga vigorsii* | Vigors's Sunbird | 4 | 0.11 | 4 | 0.48 | 0.1 | 1 | 0.03 | 0 | 0.1 | 0.88 | -1.4 | 3.43 | * | 0.3 | -0.6 | 1.5 | ** | -1.17 | -3.1 | 0.64 | ** |
| 120 | WT | *Chlidonias hybridus* | Whiskered Tern | 1 | 0.03 | 1 | 0.21 | 0 | 0.9 | 0.02 | 0 | 0.1 | -1.25 | -4.3 | 2.14 | * | 0.09 | -1.1 | 1.2 | * | -0.8 | -2.9 | 1.06 | * |
| 121 | W_BD | *Dicrurus caerulescens* | White-bellied Drongo | 2 | 0.06 | 2 | 0.34 | 0 | 1 | 0.02 | 0 | 0.1 | 0.85 | -1.5 | 3.61 | * | 0.11 | -1 | 1.1 | ** | -0.96 | -3.1 | 0.88 | ** |
| 122 | W_BF | *Rhipidura aureola* | White-browed Fantail | 5 | 0.14 | 8 | 0.37 | 0.1 | 0.9 | 0.07 | 0 | 0.2 | -0.24 | -2 | 1.86 | * | 0.46 | -0.4 | 1.7 | ** | -0.94 | -2.7 | 0.48 | ** |
| 123 | W_CB | *Megalaima viridis* | White-cheeked Barbet | 4 | 0.11 | 8 | 0.07 | 0 | 0.6 | 0.07 | 0 | 0.3 | 1.95 | 0.24 | 4.08 | *** | 0.27 | -0.7 | 1.4 | ** | -0.53 | -2.6 | 1.36 | * |
| 124 | W_EB | *Pycnonotus leucotis* | White-eared Bulbul | 6 | 0.17 | 19 | 0.1 | 0 | 0.3 | 0.21 | 0.1 | 0.4 | -2.64 | -5.2 | -0.5 | *** | 0.36 | -0.4 | 1.3 | ** | -1.08 | -2.3 | 0.04 | ** |
| 125 | W_EBZ | *Butastur teesa* | White-eyed Buzzard | 1 | 0.03 | 1 | 0.23 | 0 | 0.9 | 0.02 | 0 | 0.1 | -0.67 | -4.1 | 2.62 | * | 0.06 | -1.1 | 1.1 | * | -0.91 | -3 | 1.06 | * |
| 126 | W_RM | *Lonchura striata* | White-rumpedMunia | 3 | 0.09 | 4 | 0.31 | 0 | 0.9 | 0.03 | 0 | 0.1 | -0.09 | -2.1 | 2.7 | * | 0.35 | -0.6 | 1.6 | ** | -1.11 | -3.1 | 0.75 | ** |
| 127 | W_RS | *Copsychus malabaricus* | White-rumpedShama | 6 | 0.17 | 13 | 0.17 | 0 | 0.8 | 0.07 | 0 | 0.2 | 2.26 | 0.34 | 4.63 | *** | 0.25 | -0.7 | 1.3 | ** | -0.82 | -2.9 | 0.98 | ** |
| 128 | W_SF | *Rhipidura albogularis* | White-spotted Fantail | 2 | 0.06 | 2 | 0.3 | 0 | 1 | 0.02 | 0 | 0.1 | -1.32 | -4.5 | 1.69 | * | 0.06 | -1.1 | 1.1 | * | 0.39 | -1.4 | 2.42 | * |
| 129 | W_TK | *Halcyon smyrnensis* | White-throated Kingfisher | 12 | 0.34 | 20 | 0.52 | 0.2 | 0.9 | 0.06 | 0 | 0.1 | -2.55 | -5.4 | -0 | *** | 0.36 | -0.6 | 1.5 | ** | 0 | -1.4 | 1.64 | * |
| 130 | W_TS | *Hirundo smithii* | Wire-tailed Swallow | 4 | 0.11 | 4 | 0.72 | 0.2 | 1 | 0.02 | 0 | 0.1 | -0.68 | -3.7 | 2.58 | * | -0.02 | -1.3 | 1 | * | -0.6 | -2.6 | 1.38 | * |
| 131 | WS | *Tringa glareola* | Wood Sandpiper | 1 | 0.03 | 1 | 0.14 | 0 | 0.9 | 0.02 | 0 | 0.1 | -1.16 | -4.3 | 1.86 | * | 0.35 | -0.6 | 1.6 | ** | -0.96 | -3 | 0.85 | ** |
| 132 | Y_EB | *Chrysomma sinense* | Yellow-eyed Babbler | 6 | 0.17 | 7 | 0.4 | 0.1 | 1 | 0.04 | 0 | 0.1 | -2.23 | -5.1 | 0.5 | ** | 0.4 | -0.5 | 1.6 | ** | 0.17 | -1.5 | 1.86 | * |
| 133 | Y_FGP | *Treron phoenicopterus* | Yellow-footed Green-pigeon | 4 | 0.11 | 4 | 0.44 | 0.1 | 0.9 | 0.03 | 0 | 0.1 | -1.7 | -4.6 | 1.77 | * | 0.19 | -0.7 | 1.2 | ** | -1.36 | -3.3 | 0.5 | ** |
| 134 | Y_FPW | *Dendrocopos mahrattensis* | Yellow-fronted Pied Woodpecker | 2 | 0.06 | 2 | 0.38 | 0 | 1 | 0.02 | 0 | 0.1 | -1.04 | -4.1 | 2.5 | * | 0.2 | -0.8 | 1.4 | ** | -1.1 | -3.2 | 0.98 | ** |
| 135 | Y_TS | *Gymnoris xanthocollis* | Yellow-throated Sparrow | 19 | 0.54 | 30 | 0.87 | 0.6 | 1 | 0.13 | 0.1 | 0.2 | -1.39 | -3.3 | 1.36 | * | 0.1 | -1 | 1.1 | * | -1.03 | -2.6 | 0.58 | ** |

Abbr – Abbreviation; Pre – Presence; Det – Detections; CI – Credible interval; *- Weak effect; ** - Moderate effect; *** - Strong effect; UI- Unidentified

1. **AMPHIBIANS**

|  |  |  |  |  |  |  | **Occupancy** | | | **Detection** | | | **Slope parameter** | | |  | **Slope parameter** | | |  | **Slope parameter** | | |  |
| --- | --- | --- | --- | --- | --- | --- | --- | --- | --- | --- | --- | --- | --- | --- | --- | --- | --- | --- | --- | --- | --- | --- | --- | --- |
|  |  |  |  |  |  |  |  |  |  |  |  |  | **Elevation (α1)** | | |  | **Forest (α2)** | | |  | **Anthropogenic habitat cover (α3)** | | |  |
| **Sr. No.** | **Abbr** | **Scientific Name** | **Common Name** | **Pre** | **Naïve occupancy** | **Det** | **Mean** | **95% CI** | | **Mean** | **95% CI** | | **Mean** | **95% CI** | |  | **Mean** | **95% CI** | |  | **Mean** | **95% CI** | |  |
| 1 | AR_bomb | *Raorchestes* cf*. bombayensis* | Bombay Bush Frog | 5 | 0.142 | 22 | 0.013 | 0.001 | 0.12 | 0.063 | 0.018 | 0.182 | 2.431 | 0.83 | 4.743 | *** | 1.427 | -0.191 | 3.328 | ** | -0.681 | -2.29 | 0.824 | ** |
| 2 | AR_gha | *Raorchestes ghatei* | Ghate's Bush Frog | 2 | 0.057 | 8 | 0.009 | 0 | 0.105 | 0.064 | 0.014 | 0.283 | 1.691 | -0.051 | 4.096 | ** | 1.297 | -0.476 | 3.517 | ** | -0.69 | -2.364 | 0.885 | ** |
| 3 | AP_mac | *Polypedates maculatus* | Common Tree Frog | 12 | 0.342 | 36 | 0.501 | 0.16 | 0.876 | 0.028 | 0.013 | 0.058 | 0.581 | -1.331 | 2.738 | * | 2.323 | 0.561 | 4.784 | *** | -0.988 | -2.67 | 0.08 | ** |
| 4 | AI_lei | *Indirana leithii* | Leith's leaping Frog | 3 | 0.085 | 11 | 0.055 | 0.001 | 0.63 | 0.004 | 0 | 0.027 | 1.685 | -0.673 | 4.685 | ** | 1.488 | -0.735 | 4.288 | ** | -0.796 | -2.538 | 0.653 | ** |
| 5 | AS_bre | *Sphaerotheca breviceps* | Indian Burrowing Frog | 5 | 0.142 | 18 | 0.21 | 0.031 | 0.749 | 0.005 | 0.001 | 0.024 | -0.526 | -3.256 | 3.573 | * | 2.416 | 0.594 | 5.251 | *** | -0.946 | -2.663 | 0.293 | ** |
| 6 | AD_mel | *Duttaphrynus melanostictus* | Asian Common Toad | 13 | 0.371 | 27 | 0.826 | 0.41 | 0.994 | 0.018 | 0.007 | 0.038 | 1.298 | -1.145 | 3.911 | * | 1.625 | -0.178 | 3.853 | ** | -0.576 | -1.787 | 0.708 | ** |
| 7 | AF_sp1 | *Fejervarya sp 1* | Cricket Frog | 19 | 0.542 | 41 | 0.948 | 0.64 | 0.999 | 0.042 | 0.025 | 0.069 | -0.005 | -2.741 | 3.182 | * | 0.814 | -1.356 | 3.295 | ** | -0.988 | -2.596 | 0.205 | ** |
| 8 | AF_sp2 | *Fejervarya sp 2* | Cricket Frog | 16 | 0.457 | 49 | 0.538 | 0.315 | 0.805 | 0.094 | 0.06 | 0.136 | 1.062 | -0.159 | 2.689 | ** | 0.239 | -0.761 | 1.511 | ** | -0.337 | -1.165 | 0.613 | ** |
| 9 | AH_bah | *Hydrophylax bahuvistara* | Fungoid Frog | 6 | 0.171 | 18 | 0.191 | 0.044 | 0.527 | 0.01 | 0.002 | 0.034 | -1.248 | -3.182 | 0.67 | ** | 0.932 | -0.327 | 2.639 | ** | -1.094 | -3.065 | -0.006 | *** |
| 10 | AU_mor | *Uperodon mormorata* | Indian Dot Frog | 4 | 0.114 | 10 | 0.054 | 0.003 | 0.402 | 0.035 | 0.011 | 0.107 | -0.532 | -2.262 | 1.901 | * | 2.161 | 0.481 | 4.55 | *** | -0.86 | -2.721 | 0.391 | ** |
| 11 | AU_glo | *Uperodon globulosus* | Balloon Frog | 1 | 0.028 | 1 | 0.052 | 0.001 | 0.836 | 0.005 | 0 | 0.053 | -0.117 | -3.079 | 3.287 | * | 1.739 | -0.193 | 4.128 | ** | -0.788 | -2.365 | 0.594 | ** |
| 12 | AE_cya | *Euphlyctis cyanophlyctis* | Skittering Frog | 14 | 0.4 | 40 | 0.786 | 0.412 | 0.98 | 0.035 | 0.019 | 0.062 | -0.936 | -3.233 | 1.408 | * | 1.289 | -0.742 | 4.013 | ** | -0.587 | -1.729 | 0.799 | ** |
| 13 | AH_tig | *Hoplobatrachus tigerinus* | Indian Bullfrog | 10 | 0.285 | 16 | 0.705 | 0.254 | 0.978 | 0.014 | 0.006 | 0.035 | 0.316 | -2.013 | 2.976 | * | 1.253 | -0.692 | 4.199 | ** | -0.845 | -2.221 | 0.175 | ** |
| 14 | AN_hum | *Nyctibatrachus humayuni* | Bombay Night Frog | 4 | 0.114 | 5 | 0.115 | 0.004 | 0.785 | 0.012 | 0.003 | 0.047 | 1.362 | -0.99 | 3.893 | ** | 1.751 | -0.266 | 4.624 | ** | -0.811 | -2.696 | 0.508 | ** |
| 15 | AI_mah | *Indotyphlus battersbyi* | Battersby's Caecilian | 1 | 0.028 | 1 | 0.062 | 0.001 | 0.963 | 0.004 | 0 | 0.047 | 1.030 | -1.395 | 3.851 | * | 1.448 | -0.85 | 4.276 | ** | -0.703 | -2.307 | 0.89 | ** |
| 16 | AM_orn | *Microhyla ornata* | Ornate Narrow Mouthed Frog | 4 | 0.114 | 7 | 0.123 | 0.023 | 0.54 | 0.016 | 0.003 | 0.063 | -0.164 | -2.213 | 1.601 | * | -0.985 | -3.224 | 0.855 | ** | -0.678 | -1.93 | 0.374 | ** |

Abbr – Abbreviation; Pre – Presence; Det – Detections; CI – Credible interval; *- Weak effect; ** - Moderate effect; *** - Strong effect

1. **REPTILES**

|  |  |  |  |  |  |  | **Occupancy** | | | **Detection** | | | **Slope parameter** | | |  | **Slope parameter** | | |  | **Slope parameter** | | |  |
| --- | --- | --- | --- | --- | --- | --- | --- | --- | --- | --- | --- | --- | --- | --- | --- | --- | --- | --- | --- | --- | --- | --- | --- | --- |
|  |  |  |  |  |  |  |  |  |  |  |  |  | **Elevation (α1)** | | |  | **Forest (α2)** | | |  | **Anthropogenic habitat cover (α3)** | | |  |
| **Sr. No.** | **Abbr** | **Scientific Name** | **Common Name** | **Pre** | **Naïve occupancy** | **Det** | **Mean** | **95% CI** | | **Mean** | **95% CI** | | **Mean** | **95% CI** | |  | **Mean** | **95% CI** | |  | **Mean** | **95% CI** | |  |
| 1 | RH_mur | *Hemidactylus* cf*. murrayi* | Murray's House Gecko | 28 | 0.8 | 253 | 0.973 | 0.851 | 0.998 | 0.31 | 0.272 | 0.35 | 1.706 | -0.121 | 4.684 | ** | -2.214 | -4.407 | -0.232 | *** | -2.821 | -5.034 | -1.058 | *** |
| 2 | RH_mac | *Hemidactylus maculatus* | Rock Gecko | 13 | 0.371 | 25 | 0.801 | 0.179 | 0.996 | 0.03 | 0.018 | 0.05 | 1.245 | -1.411 | 4.469 | * | 0.046 | -2.805 | 3.497 | * | -3.479 | -6.622 | -0.863 | *** |
| 3 | RH_les | *Hemidactylus leschenaultii* | Leschenault’s Bark Gecko | 8 | 0.229 | 11 | 0.882 | 0.148 | 1 | 0.011 | 0.005 | 0.024 | 1.085 | -1.777 | 4.313 | * | -0.38 | -3.497 | 3.066 | * | -3.161 | -6.501 | -0.203 | *** |
| 4 | RH_fre | *Hemidactylus frenatus* | House Gecko | 3 | 0.086 | 4 | 0.216 | 0.004 | 1 | 0.023 | 0.002 | 0.138 | -0.089 | -3.066 | 2.564 | * | -1.388 | -4.949 | 1.336 | * | 0.746 | -3.344 | 3.437 | * |
| 5 | RC_dec | *Cyrtodactylus deccanensis* | Deccan Ground Gecko | 14 | 0.4 | 31 | 0.912 | 0.298 | 0.999 | 0.026 | 0.015 | 0.042 | 1.12 | -1.525 | 4.438 | * | 0.204 | -2.726 | 3.256 | * | -3.531 | -6.944 | -0.783 | *** |
| 6 | RO_jer | *Ophisops jerdonii* | Jerdon's Snake Eye | 10 | 0.286 | 19 | 0.726 | 0.196 | 0.995 | 0.023 | 0.012 | 0.045 | 1.798 | -0.484 | 4.597 | ** | -2.101 | -4.806 | 0.956 | ** | -2.086 | -4.896 | 0.593 | ** |
| 7 | RR_lin | *Riopa lineata* | Lined Writhing Skink | 16 | 0.457 | 26 | 0.895 | 0.271 | 0.998 | 0.027 | 0.016 | 0.044 | 1.327 | -1.367 | 4.72 | * | 0.132 | -2.638 | 3.162 | * | -3.713 | -6.981 | -1.051 | *** |
| 8 | RE_car | *Eutropis carinata* | Many Keeled Grass Skink | 23 | 0.657 | 57 | 0.994 | 0.877 | 1 | 0.056 | 0.041 | 0.076 | 1.021 | -1.569 | 4.104 | * | -0.913 | -4.032 | 2.442 | * | -1.979 | -4.87 | 0.855 | ** |
| 9 | RE_all | *Eutropis allapalensis* | - | 17 | 0.486 | 33 | 0.993 | 0.807 | 1 | 0.03 | 0.019 | 0.046 | 0.706 | -1.941 | 3.774 | * | -0.422 | -3.708 | 3.224 | * | -1.902 | -4.853 | 0.884 | ** |
| 10 | RE_mac | *Eutropis macularia* | Bronze Skink | 25 | 0.714 | 147 | 0.968 | 0.792 | 0.998 | 0.162 | 0.131 | 0.197 | 1.751 | -0.525 | 4.883 | ** | -2.14 | -4.725 | 0.959 | ** | -2.89 | -5.393 | -0.975 | *** |
| 11 | RC_ver | *Calotes versicolor* | Garden Lizard | 26 | 0.743 | 72 | 0.994 | 0.875 | 1 | 0.078 | 0.06 | 0.1 | 0.596 | -2.059 | 3.728 | * | -1.154 | -4.081 | 1.947 | * | -1.672 | -4.482 | 1.02 | * |
| 12 | RM_rou | *Monilesaurus rouxii* | Roux’s Forest Lizard | 14 | 0.4 | 49 | 0.527 | 0.074 | 0.969 | 0.065 | 0.039 | 0.102 | 0.949 | -1.546 | 4.582 | * | 1.161 | -1.698 | 4.21 | * | -2.985 | -6.061 | -0.678 | *** |
| 13 | RC_zyl | *Chamaeleo zylanicus* | Indian Chameleon | 1 | 0.029 | 1 | 0.727 | 0.012 | 1 | 0.002 | 0 | 0.018 | 0.509 | -2.753 | 4.136 | * | -0.565 | -4.199 | 2.927 | * | -2.539 | -6.048 | 0.92 | ** |
| 14 | RV_ben | *Varanus benghalensis* | Monitor Lizard | 6 | 0.171 | 8 | 0.849 | 0.169 | 0.999 | 0.011 | 0.005 | 0.027 | -0.291 | -3.495 | 3.372 | * | -1.741 | -4.867 | 1.535 | * | -2.433 | -5.555 | 0.654 | ** |
| 15 | RS_spi | *Sitana spinaecephalus* | Fan-throated lizard | 10 | 0.286 | 11 | 0.778 | 0.14 | 0.999 | 0.017 | 0.007 | 0.037 | 0.094 | -2.825 | 3.116 | * | -3.087 | -6.227 | 0.365 | ** | -1.34 | -3.817 | 1.388 | * |
| 16 | RT_gra | *Trimeresurus gramineus* | Bamboo Pit Viper | 11 | 0.314 | 21 | 0.757 | 0.102 | 0.998 | 0.021 | 0.011 | 0.04 | 1.164 | -1.383 | 4.338 | * | 0.599 | -2.605 | 4.156 | * | -3.172 | -6.382 | -0.523 | *** |
| 17 | RA_nas | *Ahaetulla nasuta* | Green Vine Snake | 11 | 0.314 | 25 | 0.895 | 0.185 | 0.999 | 0.018 | 0.009 | 0.035 | 1.034 | -1.696 | 4.383 | * | 0.344 | -2.633 | 3.447 | * | -3.237 | -6.598 | -0.308 | *** |
| 18 | RB_cae | *Bungarus caeruleus* | Common Krait | 7 | 0.2 | 8 | 0.988 | 0.513 | 1 | 0.008 | 0.003 | 0.017 | 0.643 | -2.099 | 3.835 | * | -0.434 | -3.577 | 2.99 | * | -2.001 | -5.266 | 1.239 | * |
| 19 | RD_tri | *Dendrelaphis tristis* | Common Bronze Back Tree Snake | 5 | 0.143 | 8 | 0.664 | 0.048 | 0.998 | 0.011 | 0.004 | 0.027 | -0.782 | -3.791 | 2.966 | * | -0.813 | -4.021 | 2.417 | * | -2.905 | -6.229 | 0.129 | ** |
| 20 | RP_muc | *Ptyas mucosus* | Indian Rat Snake | 10 | 0.286 | 10 | 0.985 | 0.609 | 1 | 0.011 | 0.005 | 0.022 | -0.505 | -3.992 | 3.187 | * | -1.546 | -4.842 | 1.829 | * | -1.55 | -4.501 | 1.446 | * |
| 21 | RA_sto | *Amphiesma stolatum* | Buff-striped Keelback | 4 | 0.114 | 4 | 0.609 | 0.023 | 0.998 | 0.007 | 0.002 | 0.022 | 0.486 | -2.718 | 4.019 | * | -1.902 | -4.97 | 1.666 | * | -2.704 | -5.874 | 0.403 | ** |
| 22 | RO_arn | *Oligodon arnensis* | Banded Kukri | 3 | 0.086 | 3 | 0.967 | 0.179 | 1 | 0.004 | 0.001 | 0.014 | 0.807 | -2.039 | 3.707 | * | -1.193 | -4.542 | 2.099 | * | -1.561 | -4.92 | 1.734 | * |
| 23 | RO_tae | *Oligodon taeniolatus* | Russell's Kukri | 1 | 0.029 | 1 | 0.575 | 0.003 | 1 | 0.003 | 0 | 0.018 | 1.203 | -1.532 | 4.244 | * | -0.387 | -3.923 | 3.111 | * | -2.262 | -5.676 | 1.073 | * |
| 24 | RL_aul | *Lycodon aulicus* | Wolf Snake | 8 | 0.229 | 9 | 0.921 | 0.178 | 1 | 0.009 | 0.004 | 0.019 | 0.816 | -2.074 | 4.258 | * | 0.035 | -3.083 | 3.386 | * | -2.262 | -5.676 | 1.073 | * |
| 25 | RF_pis | *Fowlea piscator* | Checkered Keelback | 4 | 0.114 | 9 | 0.282 | 0.007 | 0.997 | 0.016 | 0.005 | 0.061 | 0.833 | -1.168 | 3.797 | * | 0.071 | -2.755 | 2.629 | * | -3.137 | -6.46 | -0.037 | *** |
| 26 | RN_naj | *Naja naja* | Indian Cobra | 1 | 0.029 | 2 | 0.417 | 0.002 | 0.999 | 0.005 | 0.001 | 0.046 | 0.231 | -2.922 | 3.938 | * | -0.026 | -3.511 | 3.111 | * | -2.489 | -5.72 | 0.371 | ** |
| 27 | REc_car | *Echis carinatus* | Saw scaled Viper | 4 | 0.114 | 5 | 0.861 | 0.088 | 1 | 0.006 | 0.002 | 0.017 | 0.204 | -3.299 | 4.322 | * | -0.447 | -3.67 | 2.907 | * | -2.431 | -5.822 | 0.728 | ** |
| 28 | RD_rus | *Daboia russelii* | Russel's Viper | 5 | 0.143 | 7 | 0.862 | 0.072 | 1 | 0.008 | 0.003 | 0.021 | 0.963 | -1.726 | 4.067 | * | -0.465 | -3.551 | 2.912 | * | -2.882 | -6.32 | 0.255 | ** |
|  |  |  |  |  |  |  | **Occupancy** | | | **Detection** | | | **Slope parameter** | | |  | **Slope parameter** | | |  | **Slope parameter** | | |  |
|  |  |  |  |  |  |  |  |  |  |  |  |  | **Elevation (α1)** | | |  | **Forest (α2)** | | |  | **Anthropogenic habitat cover (α3)** | | |  |
| **Sr. No.** | **Abbr** | **Scientific Name** | **Common Name** | **Pre** | **Naïve occupancy** | **Det** | **Mean** | **95% CI** | | **Mean** | **95% CI** | | **Mean** | **95% CI** | |  | **Mean** | **95% CI** | |  | **Mean** | **95% CI** | |  |
| 29 | RB_bed | *Boiga beddomei* | Beddome's Cat Snake | 4 | 0.114 | 7 | 0.305 | 0.004 | 0.998 | 0.011 | 0.003 | 0.049 | 1.236 | -1.079 | 4.162 | * | 0.602 | -2.927 | 3.858 | * | -2.972 | -6.424 | 0.196 | ** |
| 30 | RE_joh | *Eryx johnii* | John's Sand Boa | 1 | 0.029 | 1 | 0.603 | 0.006 | 0.999 | 0.003 | 0 | 0.026 | 0.442 | -2.735 | 3.897 | * | -1.242 | -4.68 | 2.282 | * | -2.339 | -5.779 | 0.755 | ** |
| 31 | RE_con | *Eryx conicus* | Common Sand Boa | 2 | 0.057 | 2 | 0.833 | 0.026 | 1 | 0.003 | 0.001 | 0.016 | 0.262 | -3.298 | 4.095 | * | -0.476 | -3.6 | 2.682 | * | -2.332 | -5.773 | 0.95 | ** |
| 32 | RU_mac | *Uropeltis macrolepis* | Large-scaled Shieldtail | 1 | 0.029 | 4 | 0.008 | 0 | 0.207 | 0.07 | 0.008 | 0.278 | 1.15 | -0.574 | 3.429 | ** | -0.41 | -2.643 | 1.906 | * | -2.704 | -6.147 | 0.415 | ** |
| 33 | RC_hel | *Coelognathus helena monticollaris* | Montane Trinket | 2 | 0.057 | 2 | 0.858 | 0.036 | 1 | 0.003 | 0.001 | 0.012 | 1.018 | -1.872 | 4.202 | * | -0.492 | -3.87 | 3.142 | * | -1.676 | -4.701 | 1.059 | * |
| 34 | RR_bra | *Indotyphlops braminus* | Blind snake | 1 | 0.029 | 1 | 0.86 | 0.012 | 1 | 0.003 | 0 | 0.03 | 0.242 | -3.034 | 3.675 | * | -1.38 | -5.029 | 2.162 | * | -2.625 | -6.077 | 0.654 | ** |
| 35 | RP_mol | *Python molurus* | Indian Rock Python | 2 | 0.057 | 2 | 0.97 | 0.112 | 1 | 0.003 | 0.001 | 0.013 | 0.066 | -3.455 | 3.423 | * | -0.653 | -3.815 | 2.535 | * | -1.006 | -4.535 | 2.546 | * |
| 36 | RC_rhy | *Cerberus rynchops* | Dog-faced Water Snake | 2 | 0.057 | 2 | 0.949 | 0.057 | 1 | 0.003 | 0.001 | 0.024 | -0.045 | -3.454 | 3.449 | * | -0.573 | -3.583 | 2.474 | * | -1.58 | -4.866 | 1.758 | * |

Abbr – Abbreviation; Pre – Presence; Det – Detections; CI – Credible interval; *- Weak effect; ** - Moderate effect; *** - Strong effect
